# Genotypic and Allelic Frequencies of Degenerative Myelopathy in an Italian Canine Population

**DOI:** 10.3390/ani14182712

**Published:** 2024-09-19

**Authors:** Sara Ghilardi, Giulietta Minozzi, Maria Grazia De Iorio, Camilla Gonzi, Stefano Frattini, Mara Bagardi, Paola G. Brambilla, Alessandra Paganelli, Michele Polli

**Affiliations:** 1 Department of Veterinary Medicine and Animal Sciences—DIVAS, University of Milan, 26900 Lodi, Italy; sara.ghilardi@unimi.it (S.G.); giulietta.minozzi@unimi.it (G.M.); maria.deiorio@unimi.it (M.G.D.I.); gonzicamilla9@gmail.com (C.G.); mara.bagardi@unimi.it (M.B.); michele.polli@unimi.it (M.P.); 2 Vetogene Laboratory—ENCI Servizi SRL, 20139 Milan, Italy; sfrattini.vetogene@enciservizi.it (S.F.); apaganelli.vetogene@enciservizi.it (A.P.)

**Keywords:** canine degenerative myelopathy, dog breeds, genetic test, allele frequency

## Abstract

**Simple Summary:**

Canine degenerative myelopathy is a fatal neurodegenerative disorder that affects the spinal cord. It is a late-onset disease, with the first clinical signs becoming evident later in life at approximately 8 years of age. The aim of this study was to retrospectively evaluate allelic and genotypic frequencies of the c.118G > A and c.52A > T mutations in an Italian canine population of 1667 dogs belonging to 84 breeds. The results showed that 65.47% (n. 893) of the subjects were clear, 25.59% (n. 349) were heterozygous carriers, and 8.94% (n. 122) were homozygous for the risk allele. The highest frequency was observed in Pembroke Welsh Corgis (55.49%).

**Abstract:**

Canine degenerative myelopathy is a fatal neurodegenerative disorder that affects the spinal cord. It is a late-onset disease, with symptoms becoming evident later in life at approximately 8 years of age. The principal aim of this study was to retrospectively evaluate allelic and genotypic frequencies of the c.118G > A and c.52A > T mutations located on the *SOD1* gene in an Italian canine population to provide detailed information on the prevalence of the mutations in the country. The genetic data of different breeds were collected through DNA tests over a nine-year period in the Italian canine population. For each dog, the breed, sex, age, and DNA test results were recorded. Allelic and genotypic frequencies were calculated. A total of 1667 DNA tests for the c.118G > A and c.52A > T mutations were carried out on 84 breeds. For the analysis of prevalence, only breeds counting more than 20 subjects have been considered, for a total of 1410 DNA tests obtained from 13 different breeds. In the population tested for the c.118G > A mutation, 65.47% (n. 893) of the subjects were clear, 25.59% (n. 349) were heterozygous carriers, and 8.94% (n. 122) were homozygous for the mutated allele. The mutation showed the highest frequency in Pembroke Welsh Corgis (55.49%) and the lowest frequencies in Poodles (6.32%) and Australian Shepherds (7.14%). The allelic frequency of the c.52A > T mutation was 7.61% in the Bernese Mountain dog. Neither variant differed between females and males in genotypic frequencies. The present study provides insights into the allelic and genotypic frequencies of canine degenerative myelopathy in different dog breeds in Italy.

## 1. Introduction

Canine degenerative myelopathy (DM) is a fatal neurodegenerative disease that affects the spinal cord, similar to forms of amyotrophic lateral sclerosis (ALS) in humans [1,2,3,4]. In dogs, the disease was first recognized in German Shepherds around 40 years ago [5]; however, it has been diagnosed in a large number of breeds ever since, the most common being the Pembroke Welsh Corgi [6], the Boxer [7], the Chesapeake Bay Retriever, and the Rhodesian Ridgeback [2]. Canine degenerative myelopathy is a late-onset disease, with the first clinical signs becoming evident later in life at approximately 8 years of age. No sex predilection has been shown [1,2,3,4,5]. Signs include general proprioceptive ataxia and spastic paraparesis, which then evolve into paraplegia, thoracic limb weakness, and flaccid tetraplegia. After the occurrence of clinical signs, the disease could last even longer than three years; however, most owners prefer to proceed with euthanasia when the dog shows paraplegia [1,2,8]. Diagnosis of DM can be accomplished through histopathologic observation of the spinal cord, which will present signs of axonal and myelin degeneration. For this reason, the definitive diagnosis is only reached postmortem [9,10,11]. From a genetic point of view, DM is an autosomal recessive disease, with an age-related incomplete penetrance: affected dogs are homozygous for the superoxide dismutase 1 (*SOD1*) gene mutation c.118G > A [2]. This mutation has been identified in several different breeds [12], while another *SOD1* gene variant, the c.52A > T, has been reported only in the Bernese Mountain dog so far [12,13,14]. Since not all homozygous dogs develop symptoms, the main hypothesis is that additional genetic factors may play a role in the onset and the course of the disease [15]. In fact, a study on homozygous DM-affected and non-affected Pembroke Welsh Corgis reported the discovery of a modifier locus on the canine chromosome 25: a haplotype within the *SP110* nuclear body protein. This haplotype is associated with an increased probability for homozygous dogs of developing DM [15]. To date, the pathophysiology of DM remains unclear [2,6]. Given this information, the principal aim of this study was to retrospectively evaluate the allelic and genotypic frequencies of the *c.118G > A* and *c.52A > T* mutations in an Italian canine population over a nine-year period, in order to provide the prevalence of the mutation in the country. This information could be useful for setting an appropriate breeding scheme aimed at lowering the frequency of the mutation in the Italian canine population.

## 2. Materials and Methods

### 2.1. Population Enrollment

This was a retrospective observational study conducted between 2014 and 2023. All DNA tests for *c.118G > A* detection conducted on dogs from all over Italy during this period of time were included in this study; DNA tests for *c.52A > T* detection conducted only on Bernese Mountain dogs were also included. Each subject was privately owned and DNA tests were required either by the owner/breeder or the veterinarian in order to assess the genetic status of the dog. Almost all dogs enrolled in this study belonged to private owners who decided to test the dog for future mating or to obtain indications in anticipation of a possible diagnosis. All dogs tested in the laboratory named “Vetogene Laboratory” between 2014 and 2023 have been included in this study.

Blood sampling for the DNA test execution was operated by authorized veterinarians and was accompanied by a medical certification. Blood samples were placed into EDTA tubes and stored at +4 °C for a maximum of 4–5 days prior to examination and extraction. DNA tests were conducted in a commercial laboratory, Vetogene—ENCI Servizi Laboratory, which is one of the official reference laboratories for the execution of DNA tests for the Italian Kennel Club Ente Nazionale Cinofilia Italiana (ENCI) and it is the reference laboratory for the University of Milan. For each dog, the breed, sex, age, and DNA test results were recorded (clear, heterozygous carrier, or homozygous for the mutated allele). No ethical approval was required because the data were provided by the laboratory and were collected from general clinical practice. The use of the data has been agreed upon by the owner through written consent.

### 2.2. DNA Extraction and Analysis

At Vetogene Laboratory, DNA extraction was carried out using the E.Z.N.A.^®^ Blood DNA kit (Omega Bio-tek, Norcross, GA, USA), following the manufacturer’s instructions. The samples were analyzed through real-time PCR for the identification of the *c.118G > A* and *c.52A > T* mutations. The genotyping was validated directly on the sequence by looking at the mutation using the Sanger sequencing method.

### 2.3. Statistical Analysis

Statistical analysis was conducted using the SAS 9.4 software (SAS Inc., Cary, NC, USA). The Shapiro–Wilk test was performed to assess the distribution of variables. Normally distributed variables were expressed in terms of mean and standard deviation (SD); non-normally distributed variables were described in terms of median and interquartile range (IQR); and categorical data were described by frequencies. Initially, frequencies of clear (homozygous for the wild-type allele, G/G), affected/at-risk dogs (homozygous for the mutated allele, A/A), and heterozygous carriers (G/A) were recorded according to the breed. Subsequently, only breeds that counted more than 20 subjects were considered for the analysis; for each breed, allelic and genotypic frequencies were evaluated through the PROC FREQ and PROC ALLELE analyses offered by SAS. To compare the age of the clear subjects, heterozygous carriers, and affected/at-risk dogs, the Kruskal–Wallis test for non-normally distributed data was performed. Statistical significance was set at *p* < 0.05. All the data containing information used for the analysis and the genetic tests are reported in Supplementary Table S1 (S1_Table.pdf).

## 3. Results

A total of 1667 DNA tests for c.118G > A and *c.52A > T* detection was carried out on 1621 dogs of 84 different breeds. However, only breeds counting more than 20 subjects have been considered for this study, for a total of 1410 DNA tests obtained from 13 breeds. Of the 1410 tests, 1364 were for the detection of the *c.118G > A* mutation, while 46 were for the detection of the *c.52A > T* variant and were performed only on Bernese Mountain dogs. The 46 Bernese Mountain dogs that were tested for the *c.52A > T* mutation were also tested for the c.118G > A mutation. Absolute and relative frequencies of clear subjects, heterozygous carriers, and affected/at-risk dogs for the *c.118G > A* mutation are reported in Table 1. All the raw data used in this study are available as Supporting Information Table S1 (S1_Table.pdf).

In 65.47% of the dogs tested for the c.118G > A mutation, the test result was clear (*n* = 893), while 25.59% of the tested dogs were heterozygous carriers (*n* = 349) and 8.94% were deemed affected/at risk (*n* = 122). Interestingly, five out of the thirteen analyzed breeds (Pembroke Welsh Corgi, Hovawart, Scotch collie, Borzoi, and Cavalier King Charles Spaniel) presented percentages of heterozygous carriers plus affected/at-risk dogs higher than 50%, showing a great diffusion of the *c.118G > A* mutation in the population. Particularly, results showed that the Pembroke Welsh Corgi was the breed with the highest frequency of the DM mutation, with 42.86% *(n* = 39) of the subjects being heterozygous carriers and 34.06% (*n* = 31) being affected/at risk; thus, subjects with the *c.118G > A* mutation outnumbered clear subjects (23.08%; *n* = 21). In Borzois and Cavalier King Charles Spaniels, the c.118G > A mutation has been reported in 50% and 79.17% of dogs, respectively; however, DNA tests were performed only on 26 Borzois and on 24 Cavalier King Charles Spaniels, so the results for these breeds could have been influenced by the low numbers and the herein reported percentages may differ when considering a wider population. Concerning the Bernese Mountain dog, 129 subjects were tested for the *c.118G > A* mutation, showing 29.46% heterozygous carriers (*n* = 38) and 2.32% homozygous subjects for the mutated allele (*n* = 3). On the other hand, 46 subjects were also tested for the *c.52A > T* mutation; among these samples, 85.36% were clear (*n* = 39) and 15.22% were heterozygous carriers (*n* = 7).

The allelic frequencies for the *c.118G > A and c.52A > T* mutations are reported in Table 2.

The mutated allele A showed the highest frequency in the Pembroke Welsh Corgi, with 55.49% of the dogs presenting with the *c.118G > A* mutation. The Cavalier King Charles Spaniel had a high frequency of the DM gene mutation (54.17%), but results should be evaluated considering the low number of tested subjects. The Scotch collie, the German Shepherd, and the Hovawart had *c.118G > A* mutation frequencies of 36.36%, 29.06%, and 28.38%, respectively. On the other hand, the lowest frequencies for the mutation were found in Shetland sheepdogs (6.90%), Australian Shepherds (7.14%), and Poodles (6.32%). In Bernese Mountain dogs, the *c.118G > A* mutation frequency was 17.05%, while the *c.52A > T* mutated allele (T) frequency was 7.60%.

Out of the 1621 tested dogs, 1222 had their age registered at the time of sampling. Subjects were divided according to the DNA test results; therefore, Table 3 shows the distribution of the age of dogs among the affected/at-risk, heterozygous carriers, and clear subjects.

The mean age of the homozygous subjects for the mutated allele (A) was significantly statistically different from the mean age of the clear subjects (*p* < 0.0001) and the heterozygous carriers (*p* = 0.0002). No significant difference was found between clear subjects and heterozygous carriers.

Furthermore, differences emerged between the breeds when analyzing the mean age at which the DNA test was performed: French bulldogs, Pembroke Welsh Corgis, Scotch collies, and Cavalier King Charles Spaniels at the moment of DNA testing are, on average, under two years of age. This early testing is probably attributable to the inclusion of DM in the genetic screening protocols adopted by breeders with the aim of evaluating the suitability of potential breeders and developing adequate mating programs. On the other hand, the Australian Shepherd stands out as the breed with the highest average age at testing, recording a value of 11.5 years. The White Swiss Shepherd and the German Shepherd follow with an average of 7.5 and 8.8 years, respectively. The advanced age at the time of the DNA test suggests that it was conducted in response to symptoms already present in the subjects with the aim of confirming or excluding the genetic component.

Finally, the sex was registered for 1401 dogs. Of these, 56.10% were females (*n* = 786) and 43.90% were males (*n* = 615). Genotypic frequencies did not differ between the sexes, showing 51 (6.49%) and 52 (8.46%) homozygous females and males for the mutated allele (A), respectively. In the same way, 204 females (25.95%) and 153 males (24.88%) were heterozygous carriers.

## 4. Discussion

To the authors’ knowledge, this is the first study that reports genotypic and allelic frequencies for the *c.118G > A and c.52A > T* mutations in an Italian canine population over a nine-year period. The first interesting result is the high number of DNA tests conducted in Italy during this period of time, associated with an elevated number of tested breeds. Furthermore, tests have been requested and performed on dogs at a young age, demonstrating a growing awareness of DM among breeders, owners, and clinicians. In fact, DM is a late-onset disease, so the precocity of DNA testing is of critical importance for a correct breeding program.

The reported results showed a high prevalence of the DM-associated mutation *c.118G > A,* with 25.59% of heterozygous carriers and 8.94% of homozygous dogs for the mutated allele (A). These numbers may underestimate the true prevalence of the mutation since some symptomatic dogs could have not been tested.

Our results reported the highest frequency for the *c.118G > A* mutation in the Pembroke Welsh Corgi (55.49%). This is in line with what is already reported in the genetic study by Donner et al. conducted on over one million dogs, where allelic frequency for DM in 4364 Pembroke Welsh Corgis was 53.28% [16]. Furthermore, Zeng et al., in their study on 35,359 dogs, identified the Pembroke Welsh Corgi as the breed with the second highest frequency of the mutated allele (79%), with 3209 dogs tested for the mutation [12]. In the same way, the frequency of the mutated allele for the *c.118G > A* mutation was 69% for the Pembroke Welsh Corgi in Japan [17]. All these results confirm that this breed is one of the most representative of the DM mutation globally. These results should indeed be disseminated to all relevant associations in order to increase awareness and create selective breeding strategies.

In our study, the allelic frequency for the *c.118G > A* mutation was 54.17% for Cavalier King Charles Spaniels. Although we are aware that one of the limits of these results lies in the fact that our population counted only 24 dogs for this breed, these results are comparable to those reported by Donner et al., where the allelic frequency in this breed was 51.03% in 2242 tested dogs [15], and by Zeng et al., where the allelic frequency was 68% (73 dogs) [12]. Similar considerations can also be made for Scotch collies, German Shepherds, and Hovawarts: according to our results, the frequencies of the mutated allele in Italy were 36.36%, 29.06%, and 28.38%, respectively. Zeng et al. reported allelic frequencies of 39%, 37%, and 38%, respectively, in much larger populations [12]. In the same way, in 2014 Holder et al. reported an allelic frequency of 38% in German Shepherds in the United Kingdom [18]. On the other hand, Donner et al. reported inferior allelic frequency for the mutation in German Shepherds: 20.42% on 15,645 tested dogs [16].

Discrepancies between our results and Zeng et al.’s results [12] can be found for the Shetland sheepdog. In fact, according to our results, allelic frequencies in Italy were very low, specifically 6.9% for the 29 tested dogs. Zeng et al. reported a higher allelic frequency, specifically 21% for 58 tested dogs [12]. However, results from the present study are in line with what has been found by Donner et al., who reported an allelic frequency of 9.10% for 945 subjects [16]. Overall, the frequency is not so high when compared to all the other breeds, and the results obtained by Zeng et al. may be related to their specific population.

Furthermore, a great difference concerns allelic frequencies in Australian Shepherds: our results showed a frequency of 7.14% for the mutated allele, while Zeng et al. found a frequency of 41% in the same breed [12]. Again, results from our population are similar to those of the Donner et al. study, where the allelic frequency in a population of 2296 Australian Shepherds was 8.95% [16]. Presumably, differences between our results and Zeng et al.’s results [12] can be attributed to the different popularity and breeding strategies of Australian Shepherds and Shetland sheepdogs in Italy and the United States of America, where the population of their study was selected. 

Table 4 summarizes the allelic frequencies of the mutated allele associated with DM reported in the literature for different breeds.

Results from the present study showed that the DNA tests for the *SOD1* mutation detect clear subjects and heterozygous carriers at a younger age in comparison to homozygous dogs for the (A) allele. The main hypothesis is that Italian breeders are very accurate and keen in running the genetic tests for the detection of the genetic status of their breeding dogs, so heterozygous carriers and clear subjects are identified at a young age. On the contrary, affected dogs are subjected to the DNA test later in life, presumably when symptoms become evident; consequently, the test is used as a diagnostic tool.

Finally, our results showed that genotypic frequencies for DM are not influenced by gender, confirming the absence of sex predisposition to the disease, as already reported in the literature by Nakata et al. [25]. This information is very useful as it gives more freedom on both male and female sides to define breeding strategies. We must therefore state that the population enrolled in this study was made of 56.10% females and 43.90% males. This discrepancy can be associated with the fact that females are bred to a greater extent than males.

Given the extent of the knowledge about DM in several dog breeds [3,6,26,27] and the advance of new technologies, further studies are looking at the variability in the expression of microRNAs (miRNAs) in the disease [3,25,28]. Nakata et al. [25] analyzed the miRNA profile of the spinal cord in DM-affected dogs and identified 3 up-regulated miRNAs and 18 down-regulated miRNAs that may influence the progression of degenerative processes. Indeed, further studies on miRNAs, combined with mutation detection, can be ideal for the detection and analysis of late-onset diseases in many species [29,30].

## 5. Conclusions

In conclusion, the present study provides insights into the allelic and genotypic frequencies of DM in different breeds in Italy over a long time period through a retrospective study. Our results showed that the allelic frequencies of the mutated allele (A) are the highest in the Pembroke Welsh Corgi breed; therefore, particular attention should be paid by the breeders of this breed in order to lower the prevalence of the c.118G > A mutation. In addition, Cavalier King Charles Spaniels, Scotch collies, German Shepherds, and Hovawarts also showed moderately high frequencies of the allele (A). Results suggest that DNA tests in Italy are an increasingly widespread diagnostic tool not only for older and symptomatic dogs but especially for younger reproducers. In general, the results suggest that Italian breeders have become more aware of the problems related to genetically transmitted, hereditary diseases. Compared to the past, we have observed an increase in the frequency of the usage of genetic tests, indicating significant progress in the management of the genetic health of dogs in breeding programs. Further research should be carried out on these late-onset pathologies, including—when possible—microRNA studies as a complementary approach.

## Figures and Tables

**Table 1:** Absolute and relative frequencies of clear subjects, heterozygous carriers, and affected/at-risk Italian dogs for the c.118G > A mutation. Only breeds that counted more than 20 subjects were considered for the analysis.

Breed	G/G		G/A		A/A	
	N	%	N	%	N	%
German Shepherd	147	55.47%	82	30.94%	36	13.59%
Czechoslovakian wolfdog	142	68.93%	56	27.18%	8	3.89%
Poodle	153	87.93%	20	11.49%	1	0.58%
Australian Shepherd	133	86.36%	20	12.99%	1	0.65%
French bulldog	83	62.40%	41	30.83%	9	6.77%
Pembroke Welsh Corgi	21	23.08%	39	42.86%	31	34.06%
White Swiss Shepherd	51	80.96%	10	15.87%	2	3.17%
Hovawart	18	48.65%	17	45.94%	2	5.41%
Scotch collie	14	42.42%	14	42.42%	5	15.16%
Shetland sheepdog	25	86.21%	0	0.00%	4	13.79%
Borzoi	13	50.00%	0	0.00%	13	50.00%
Cavalier King Charles Spaniel	5	20.83%	12	50.00%	7	29.17%
Bernese Mountain dog	88	68.22%	38	29.46%	3	2.32%
Total	893	65.47%	349	25.59%	122	8.94%

**Table 2:** Allelic frequencies for the mutated and wild-type alleles (A and G, respectively) for the *c.118G > A* mutation divided according to the breed, and allelic frequencies for the mutated and wild-type alleles (T and A, respectively) for the *c.52A > T* mutation in the Bernese Mountain dog. For each frequency, the confidence interval is reported.

Breed	A		G	
	Frequency	CI	Frequency	CI
German Shepherd (*n* = 265)	0.2906	0.2472–0.3340	0.7094	0.6660–0.7528
Czechoslovakian wolfdog (*n* = 206)	0.1748	0.1383–0.2136	0.8252	0.7864–0.8617
Poodle (*n* = 174)	0.0632	0.0374–0.0891	0.9368	0.9109–0.9626
Australian Shepherd (*n* = 154)	0.0714	0.0455–0.1006	0.9286	0.8994–0.9545
French bulldog (*n* = 133)	0.2218	0.1692–0.2744	0.7782	0.7256–0.8308
Pembroke Welsh Corgi (*n* = 91)	0.5549	0.4780–0.6319	0.4451	0.3681–0.5220
White Swiss Shepherd (*n* = 63)	0.1111	0.0556–0.1746	0.8889	0.8254–0.9444
Hovawart (*n* = 37)	0.2838	0.1892–0.3784	0.7162	0.6216–0.8108
Scotch collie (*n* = 33)	0.3636	0.2424–0.4848	0.6364	0.5152–0.7576
Shetland sheepdog (*n* = 29)	0.0690	0.0172–0.1379	0.9310	0.8621–0.9828
Borzoi (*n* = 26)	0.2500	0.1538–0.3462	0.7500	0.6538–0.8462
Cavalier King Charles Spaniel (*n* = 24)	0.5417	0.3958–0.6875	0.4583	0.3125–0.6042
Bernese Mountain dog (*n* = 129)	0.1705	0.1279–0.2171	0.8295	0.7829–0.8721
	**T**		**A**	
	**Frequency**	**CI**	**Frequency**	**CI**
Bernese Mountain dog (*n* = 46)	0.0760	0.0338–0.1556	0.9239	0.8444–0.9662

CI = confidence interval.

**Table 3:** Mean, standard deviation, and median age of 1222 dogs divided according to their genotype for the c.118G > A mutation.

	N	Mean (Days/Years)	Standard Deviation (Days/Years)	Median (Days/Years)
(G/G)	791	885/2.42	782/2.14	627/1.72
(G/A)	337	979/2.68	929/2.55	662/1.81
(A/A)	94	1715/4.70 *^,#^	1412/3.87	967/2.65

* The mean age is statistically different from the mean age of the clear subjects (*p* < 0.05). ^#^ The mean age is statistically different from the mean age of the heterozygous carriers (*p* < 0.05).

**Table 4:** Reported frequencies of the (A) allele in breeds predisposed to canine degenerative myelopathy (note: in the Bernese Mountain dog, the allelic frequency of the *c.52A > T* mutation is also reported). For each breed, the numerosity of the population, the frequency of the mutated allele, and the bibliographic references are reported.

Breed	n	Frequency of (A)	Reference
Pembroke Welsh Corgi	91	0.55	Ghilardi et al., 2024 [presented results]
	4364	0.53	Donner et al., 2023 [16]
	3209	0.79	Zeng et al., 2014 [12]
	122	0.69	Chang et al., 2013 [17]
German Shepherd dog	265	0.29	Ghilardi et al., 2024 [presented results]
	15,645	0.20	Donner et al., 2023 [16]
	6458	0.37	Zeng et al., 2014 [12]
	541	0.22	Maki et al., 2022 [19]
	150	0.38	Holder et al., 2014 [18]
	95	0.12	Santos et al., 2020 [20]
	40	0.20	Cocostîrc et al., 2023 [21]
	29	0.14	Kountourantzis et al., 2023 [22]
Collie	33	0.36	Ghilardi et al., 2024 [presented results]
	1207	0.13	Donner et al., 2023 [16]
	151	0.39	Zeng et al., 2014 [12]
	29	0.14	Kohyama et al., 2017 [23]
Cavalier King Charles Spaniel	24	0.54	Ghilardi et al., 2024 [presented results]
	2242	0.51	Donner et al., 2023 [16]
	73	0.68	Zeng et al., 2014 [12]
Australian Shepherd	154	0.07	Ghilardi et al., 2024 [presented results]
	2296	0.09	Donner et al., 2023 [16]
	113	0.41	Zeng et al., 2014 [12]
Poodle	174	0.06	Ghilardi et al., 2024 [presented results]
	4203	0.04	Donner et al., 2023 [16]
	533	0.07	Zeng et al., 2014 [12]
Hovawart	37	0.28	Ghilardi et al., 2024 [presented results]
	64	0.38	Zeng et al., 2014 [12]
Shetland sheepdog	29	0.07	Ghilardi et al., 2024 [presented results]
	945	0.09	Donner et al., 2023 [16]
	58	0.21	Zeng et al., 2014 [12]
Bernese Mountain dog c.118 G > A	129	0.17	Ghilardi et al., 2024 [presented results]
	2413	0.38	Zeng et al., 2014 [12]
	955	0.26	Donner et al., 2023 [16]
	33	0.38	Letko et al., 2023 [24]
Bernese Mountain dog c.52A > T	46	0.08	Ghilardi et al., 2024 [presented results]
	33	0.02	Letko et al., 2023 [24]

## Data Availability

All data presented in this study are available as Supporting Materials in Table S1 (S1_File.pdf).

## Supplementary Materials

The following supporting information can be downloaded at https://www.mdpi.com/article/10.3390/ani14182712/s1.
