# Supplementary material for: Genotypic and Allelic Frequencies of Degenerative Myelopathy in an Italian Canine Population
Source: Animals (Basel). 2024 Sep 19;14(18):2712. doi: 10.3390/ani14182712 (PMC11429382; doi:10.3390/ani14182712)
Supplement: Supplementary file 1 [file animals-14-02712-s001.zip › animals-3160583-supplementary.pdf]

| RECORD | Breed | DNA test result | Sex | Birthday | DNA test date | Age at the DNA test (days) |
|---|---|---|---|---|---|---|
| 26901 | Czechoslovakian wolfdog | CLEAR | M | 02/01/13 | 05/05/14 | 488 |
| 26962 | Czechoslovakian wolfdog | CARRIER | M | 02/12/12 | 15/05/14 | 529 |
| 26963 | Barzoi | CLEAR | M | 27/03/13 | 19/05/14 | 418 |
| 26964 | German shepherd | CLEAR | M | 30/11/05 | 19/05/14 | 3092 |
| 26965 | Czechoslovakian wolfdog | CLEAR | M | 12/04/12 | 20/05/14 | 768 |
| 27025 | Welsh Corgi Pembroke | AFFECTED | M | 21/09/13 | 18/05/14 | 239 |
| 27063 | Mixed breed | CARRIER | F | 16/01/02 | 22/05/14 | 4509 |
| 27067 | Czechoslovakian wolfdog | CLEAR | M | 07/01/13 | 23/05/14 | 501 |
| 27105 | German shepherd | AFFECTED | F | 16/08/03 | 27/05/14 | 3937 |
| 27106 | Dalmatian | AFFECTED | F | 20/03/03 | 29/05/14 | 4088 |
| 27107 | Czechoslovakian wolfdog | CLEAR | F | 29/06/11 | 22/05/14 | 1058 |
| 27125 | Welsh Corgi Pembroke | AFFECTED | F | 19/01/12 | 03/06/14 | 866 |
| 27126 | German shepherd | CARRIER | F | 17/01/10 | 03/06/14 | 1598 |
| 27131 | Hovawart | CARRIER | F | 25/03/12 | 27/05/14 | 793 |
| 27180 | German shepherd | CLEAR | F | 30/11/04 | 16/06/14 | 3485 |
| 27237 | German shepherd | AFFECTED | M | 12/07/05 | 17/06/14 | 3262 |
| 27294 | Hovawart | CARRIER | F | 06/04/12 | 23/06/14 | 808 |
| 27354 | German shepherd | AFFECTED | F | 01/03/03 | 01/07/14 | 4140 |
| 27418 | Czechoslovakian wolfdog | CLEAR | F | 05/01/11 | 09/07/14 | 1281 |
| 27419 | German shepherd | CARRIER | F | 05/03/10 | 04/07/14 | 1582 |
| 27425 | Bernese Mountain Dog | CARRIER | M | 21/12/08 | 23/06/14 | 2010 |
| 27426 | Bernese Mountain Dog | CARRIER | M | 28/02/11 | 09/07/14 | 1227 |
| 27567 | Dogue de Bordeaux | CLEAR | M | 24/09/08 | 05/07/14 | 2110 |
| 27610 | Czechoslovakian wolfdog | CLEAR | M | 20/11/12 | 14/07/14 | 601 |
| 27614 | Smooth collie | CLEAR | M | 03/03/08 | 17/07/14 | 2327 |
| 27615 | Smooth collie | CLEAR | F | 19/05/10 | 17/07/14 | 1520 |
| 27726 | Czechoslovakian wolfdog | CLEAR | F | 30/11/11 | 28/07/14 | 971 |
| 27774 | Barzoi | CARRIER | F | 28/06/11 | 19/08/14 | 1148 |
| 27859 | Barzoi | CLEAR | F | 14/06/14 | 01/09/14 | 79 |
| 27860 | Barzoi | CARRIER | F | 14/06/14 | 01/09/14 | 79 |
| 27861 | Czechoslovakian wolfdog | CLEAR | F | 12/11/09 | 01/09/14 | 1754 |
| 27909 | Czechoslovakian wolfdog | CLEAR | M | 05/02/12 | 10/09/14 | 948 |

| | | | | | | |
|---|---|---|---|---|---|---|
| 27910 | Czechoslovakian wolfdog | CARRIER | M | 18/10/13 | 10/09/14 | 327 |
| 27913 | Czechoslovakian wolfdog | CARRIER | F | 09/09/12 | 09/09/14 | 730 |
| 27914 | Czechoslovakian wolfdog | CARRIER | M | 31/10/12 | 08/09/14 | 677 |
| 27943 | Smooth collie | CLEAR | F | 03/03/08 | 10/09/14 | 2382 |
| 28008 | Golden retriever | CLEAR | M | 02/10/03 | 18/09/14 | 4004 |
| 28009 | Bernese Mountain Dog | CARRIER | F | 14/10/11 | 21/09/14 | 1073 |
| 28084 | Bernese Mountain Dog | CLEAR | F | 05/09/08 | 24/09/14 | 2210 |
| 28085 | Bernese Mountain Dog | CARRIER | F | 25/05/11 | 24/09/14 | 1218 |
| 28086 | Bernese Mountain Dog | CLEAR | F | 09/03/12 | 24/09/14 | 929 |
| 28087 | Bernese Mountain Dog | CLEAR | F | 09/03/12 | 24/09/14 | 929 |
| 28112 | Barzoi | CLEAR | M | 23/04/10 | 28/09/14 | 1619 |
| 28113 | Barzoi | CLEAR | M | 02/04/13 | 28/09/14 | 544 |
| 28211 | Welsh Corgi Cardigan | CLEAR | F | 07/07/12 | 20/09/14 | 805 |
| 28251 | French Bulldog | CARRIER | M | 14/05/10 | 30/09/14 | 1600 |
| 28276 | Czechoslovakian wolfdog | CLEAR | F | 06/05/13 | 11/09/14 | 493 |
| 28312 | German shepherd | AFFECTED | M | 09/02/06 | 06/10/14 | 3161 |
| 28328 | Bernese Mountain Dog | CLEAR | F | 01/09/11 | 12/10/14 | 1137 |
| 28329 | Bernese Mountain Dog | CARRIER | M | 16/04/13 | 12/10/14 | 544 |
| 28367 | Czechoslovakian wolfdog | CLEAR | M | 07/04/13 | 09/09/14 | 520 |
| 28371 | Bernese Mountain Dog | CARRIER | M | 28/04/10 | 13/10/14 | 1629 |
| 28394 | German shepherd | CLEAR | M | 07/10/13 | 14/10/14 | 372 |
| 28417 | Czechoslovakian wolfdog | CLEAR | F | 07/08/10 | 08/10/14 | 1523 |
| 28458 | Saarlos wolfdog | CLEAR | F | 05/11/13 | 20/10/14 | 349 |
| 28470 | Czechoslovakian wolfdog | CLEAR | F | 22/10/10 | 21/10/14 | 1460 |
| 28482 | Hovawart | CARRIER | M | 31/08/14 | 23/10/14 | 53 |
| 28546 | German shepherd | AFFECTED | M | 04/02/06 | 18/10/14 | 3178 |
| 28569 | Bernese Mountain Dog | CARRIER | F | 04/06/13 | 01/11/14 | 515 |
| 28774 | Czechoslovakian wolfdog | CLEAR | M | 05/06/14 | 19/11/14 | 167 |
| 28775 | Czechoslovakian wolfdog | CLEAR | F | 05/06/14 | 19/11/14 | 167 |
| 28776 | Czechoslovakian wolfdog | AFFECTED | F | 14/11/06 | 14/11/14 | 2922 |
| 28805 | Bernese Mountain Dog | CARRIER | M | 18/10/11 | 19/11/14 | 1128 |
| 28821 | Czechoslovakian wolfdog | CLEAR | F | 01/10/13 | 25/11/14 | 420 |
| 28900 | rish soft-coated Wheaten Terrie | AFFECTED | F | 09/10/12 | 02/12/14 | 784 |
| 28901 | rish soft-coated Wheaten Terrie | CLEAR | F | 03/02/13 | 02/12/14 | 667 |

| | | | | | | |
|---|---|---|---|---|---|---|
| 28910 | Czechoslovakian wolfdog | CARRIER | M | 27/10/08 | 02/12/14 | 2227 |
| 29015 | Welsh Corgi Pembroke | CARRIER | F | 21/08/10 | 02/12/14 | 1564 |
| 29069 | Bernese Mountain Dog | CARRIER | M | 02/06/10 | 13/12/14 | 1655 |
| 29076 | Rhodesian Ridgeback | CLEAR | F | 30/04/14 | 15/12/14 | 229 |
| 29078 | Bernese Mountain Dog | CLEAR | F | 27/11/12 | 14/12/14 | 747 |
| 29079 | Barzoi | CLEAR | F | 25/12/13 | 13/12/14 | 353 |
| 29089 | Bernese Mountain Dog | CARRIER | F | 03/12/13 | 14/12/14 | 376 |
| 29090 | Bernese Mountain Dog | CARRIER | M | 11/06/13 | 14/12/14 | 551 |
| 29234 | Czechoslovakian wolfdog | CLEAR | F | 07/11/13 | 01/12/14 | 389 |
| 29321 | Rhodesian Ridgeback | CLEAR | M | 06/02/13 | 16/01/15 | 709 |
| 29338 | Rhodesian Ridgeback | CLEAR | F | 25/03/04 | 16/01/15 | 3949 |
| 29416 | Czechoslovakian wolfdog | CARRIER | F | 09/01/12 | 13/01/15 | 1100 |
| 29504 | Czechoslovakian wolfdog | CARRIER | M | 24/09/13 | 22/01/15 | 485 |
| 29510 | Bernese Mountain Dog | CLEAR | M | 04/08/11 | 01/02/15 | 1277 |
| 29511 | Bernese Mountain Dog | CLEAR | F | 29/11/07 | 01/02/15 | 2621 |
| 29636 | Boston terrier | CLEAR | M | 17/05/13 | 11/02/15 | 635 |
| 29703 | Czechoslovakian wolfdog | CLEAR | M | 04/11/12 | 10/02/15 | 828 |
| 29704 | Czechoslovakian wolfdog | CARRIER | F | 26/08/14 | 08/02/15 | 166 |
| 29755 | Bernese Mountain Dog | AFFECTED | F | 27/04/11 | 19/02/15 | 1394 |
| 29811 | Czechoslovakian wolfdog | CARRIER | M | 26/12/13 | 30/12/14 | 369 |
| 29813 | Bernese Mountain Dog | CARRIER | M | 25/03/13 | 23/02/15 | 700 |
| 29841 | German shepherd | CARRIER | M | 05/06/10 | 23/02/15 | 1724 |
| 29842 | Bernese Mountain Dog | CLEAR | M | 14/03/14 | 20/02/15 | 343 |
| 29843 | Bernese Mountain Dog | CLEAR | M | 05/05/14 | 17/02/15 | 288 |
| 29844 | Bernese Mountain Dog | CARRIER | F | 14/03/14 | 20/02/15 | 343 |
| 29876 | Czechoslovakian wolfdog | CLEAR | F | 17/10/13 | 02/01/15 | 442 |
| 29951 | Hovawart | CARRIER | M | 24/06/14 | 08/03/15 | 257 |
| 29958 | Czechoslovakian wolfdog | CLEAR | F | 02/02/14 | 04/03/15 | 395 |
| 29967 | Rhodesian Ridgeback | CLEAR | F | 01/09/13 | 02/03/15 | 547 |
| 29968 | Rhodesian Ridgeback | CLEAR | M | 02/09/13 | 02/03/15 | 546 |
| 30012 | Czechoslovakian wolfdog | CLEAR | F | 06/09/12 | 09/03/15 | 914 |
| 30027 | Hovawart | CARRIER | M | 29/09/12 | 11/03/15 | 893 |
| 30042 | Mixed breed | CLEAR | F | 01/11/05 | 13/03/15 | 3419 |
| 30043 | Czechoslovakian wolfdog | CLEAR | F | 14/12/13 | 16/03/15 | 457 |

| | | | | | | |
|---|---|---|---|---|---|---|
| 30130 | Czechoslovakian wolfdog | CLEAR | F | 20/10/13 | 10/03/15 | 506 |
| 30213 | Hovawart | AFFECTED | M | 14/11/11 | 18/03/15 | 1220 |
| 30224 | Czechoslovakian wolfdog | CARRIER | M | 11/12/14 | 30/03/15 | 109 |
| 30232 | Barzoi | CARRIER | F | 14/06/14 | 25/03/15 | 284 |
| 30288 | Hovawart | CARRIER | M | 04/12/11 | 01/04/15 | 1214 |
| 30289 | Czechoslovakian wolfdog | CLEAR | M | 08/11/13 | 02/04/15 | 510 |
| 30308 | German shepherd | CLEAR | M | 10/01/12 | 12/04/15 | 1188 |
| 30309 | Barzoi | CARRIER | M | 28/05/14 | 12/04/15 | 319 |
| 30310 | Bernese Mountain Dog | CARRIER | M | 30/03/13 | 11/04/15 | 742 |
| 30311 | Barzoi | CARRIER | M | 25/12/13 | 12/04/15 | 473 |
| 30359 | Czechoslovakian wolfdog | CARRIER | M | 20/05/13 | 13/04/15 | 693 |
| 30384 | Czechoslovakian wolfdog | CARRIER | M | 25/02/15 | 11/04/15 | 45 |
| 30385 | Czechoslovakian wolfdog | CARRIER | M | 25/02/15 | 11/04/15 | 45 |
| 30386 | Czechoslovakian wolfdog | CARRIER | M | 25/02/15 | 11/04/15 | 45 |
| 30387 | Czechoslovakian wolfdog | CLEAR | M | 25/02/15 | 11/04/15 | 45 |
| 30388 | Czechoslovakian wolfdog | CLEAR | F | 25/02/15 | 11/04/15 | 45 |
| 30389 | Czechoslovakian wolfdog | CARRIER | F | 25/02/15 | 11/04/15 | 45 |
| 30390 | Czechoslovakian wolfdog | CARRIER | F | 25/02/15 | 11/04/15 | 45 |
| 30396 | Czechoslovakian wolfdog | CARRIER | M | 01/07/08 | 10/04/15 | 2474 |
| 30458 | Czechoslovakian wolfdog | CARRIER | M | 27/02/14 | 16/04/15 | 413 |
| 30461 | Rhodesian Ridgeback | CLEAR | F | 01/09/13 | 15/04/15 | 591 |
| 30545 | German shepherd | CLEAR | F | 17/03/03 | 27/04/15 | 4424 |
| 30556 | German shepherd | CARRIER | F | 27/02/14 | 28/04/15 | 425 |
| 30583 | Czechoslovakian wolfdog | CLEAR | F | 01/11/13 | 30/04/15 | 545 |
| 30584 | Czechoslovakian wolfdog | CARRIER | F | 12/02/14 | 30/04/15 | 442 |
| 30585 | Czechoslovakian wolfdog | CARRIER | M | 12/02/14 | 30/04/15 | 442 |
| 30635 | Bernese Mountain Dog | CLEAR | M | 02/04/13 | 10/05/15 | 768 |
| 30636 | Bernese Mountain Dog | CLEAR | F | 27/05/13 | 10/05/15 | 713 |
| 30637 | Bernese Mountain Dog | CARRIER | F | 12/07/14 | 10/05/15 | 302 |
| 30677 | German shepherd | CLEAR | M | 10/03/15 | 06/05/15 | 57 |
| 30688 | Czechoslovakian wolfdog | CLEAR | M | 21/11/13 | 29/04/15 | 524 |
| 30766 | Welsh Corgi Cardigan | AFFECTED | M | 03/02/14 | 13/05/15 | 464 |
| 30822 | German shepherd | CLEAR | M | 24/10/05 | 12/05/15 | 3487 |
| 31167 | Welsh Corgi Pembroke | AFFETTO | M | 26/10/11 | 15/10/15 | 1450 |

| | | | | | | |
|---|---|---|---|---|---|---|
| 33665 | Poodle | CLEAR | | | | |
| 34642 | Czechoslovakian wolfdog | | F | 14/01/13 | 14/03/16 | 1155 |
| 35019 | Czechoslovakian wolfdog | | M | 18/12/13 | 05/04/16 | 839 |
| 36262 | Czechoslovakian wolfdog | | F | 05/08/12 | 12/07/16 | 1437 |
| 36940 | German shepherd | CLEAR | F | 09/02/13 | 30/09/16 | 1329 |
| 36985 | Czechoslovakian wolfdog | CARRIER | M | 09/01/16 | 03/10/16 | 268 |
| 37466 | ova Scotia Duck Tolling Retriev | CLEAR | | | | |
| 38374 | Czechoslovakian wolfdog | | F | 14/12/16 | 02/02/17 | 50 |
| 38375 | Czechoslovakian wolfdog | | F | 14/12/16 | 02/02/17 | 50 |
| 38376 | Czechoslovakian wolfdog | | F | 14/12/16 | 02/02/17 | 50 |
| 38411 | Rough Collie | CARRIER | F | 11/07/14 | 01/02/17 | 936 |
| 38525 | Czechoslovakian wolfdog | | F | 12/10/15 | 15/02/17 | 492 |
| 39136 | German shepherd | CLEAR | F | 30/03/15 | 20/04/17 | 752 |
| 39835 | Belgian shepherd Tervueren | CLEAR | | | | |
| 41189 | Czechoslovakian wolfdog | | F | 13/05/16 | 18/09/17 | 493 |
| 42220 | Bernese Mountain Dog | | M | 09/01/15 | 15/11/17 | 1041 |
| 42271 | Dobermann Pinscher | CLEAR | F | 03/03/16 | 22/11/17 | 629 |
| 42321 | French Bulldog | CARRIER | M | 29/04/15 | 29/11/17 | 945 |
| 42761 | Dobermann Pinscher | CLEAR | M | 01/11/13 | 13/12/17 | 1503 |
| 43058 | German shepherd | CARRIER | | | | |
| 43246 | German shepherd | CLEAR | | | | |
| 43469 | German shepherd | CLEAR | M | 05/09/11 | 28/03/18 | 2396 |
| 43496 | Shetland Sheepdog | CLEAR | F | 21/09/16 | 20/03/18 | 545 |
| 43504 | Czechoslovakian wolfdog | CARRIER | M | 12/05/15 | 29/03/18 | 1052 |
| 43547 | Hovawart | CLEAR | F | 15/05/16 | 07/04/18 | 692 |
| 43548 | Hovawart | CARRIER | M | 15/03/15 | 07/04/18 | 1119 |
| 43659 | French Bulldog | CLEAR | F | 11/01/16 | 12/04/18 | 822 |
| 43660 | French Bulldog | CARRIER | M | 30/09/17 | 12/04/18 | 194 |
| 43683 | White Swiss Shepherd Dog | CLEAR | M | 13/04/13 | 15/04/18 | 1828 |
| 43767 | Bernese Mountain Dog | CARRIER | F | 31/03/15 | 19/04/18 | 1115 |
| 43776 | Bernese Mountain Dog | CARRIER | F | 19/04/14 | 19/04/18 | 1461 |
| 43844 | Australian shepherd | CLEAR | F | 14/10/16 | 19/04/18 | 552 |
| 43898 | Australian shepherd | CLEAR | M | 01/08/17 | 20/04/18 | 262 |
| 43949 | Czechoslovakian wolfdog | CARRIER | M | 06/01/16 | 26/04/18 | 841 |

| | | | | | | |
|---|---|---|---|---|---|---|
| 43960 | French Bulldog | CLEAR | M | 06/05/14 | 07/05/18 | 1462 |
| 43966 | German shepherd | CLEAR | M | 26/04/17 | 27/04/18 | 366 |
| 43986 | Miniature poodle | CLEAR | F | 03/09/17 | 13/05/18 | 252 |
| 44034 | French Bulldog | CARRIER | M | 06/11/16 | 17/05/18 | 557 |
| 44059 | White Swiss Shepherd Dog | CLEAR | M | 13/12/15 | 17/05/18 | 886 |
| 44062 | White Swiss Shepherd Dog | CLEAR | F | 02/03/17 | 17/05/18 | 441 |
| 44065 | Hovawart | CARRIER | F | 11/05/17 | 17/05/18 | 371 |
| 44068 | Czechoslovakian wolfdog | CLEAR | M | 27/10/15 | 17/05/18 | 933 |
| 44069 | Bernese Mountain Dog | CARRIER | F | 23/02/16 | 17/05/18 | 814 |
| 44070 | Bernese Mountain Dog | CLEAR | M | 22/12/12 | 17/05/18 | 1972 |
| 44137 | White Swiss Shepherd Dog | CLEAR | F | 03/06/16 | 24/05/18 | 720 |
| 44280 | Australian shepherd | CLEAR | F | 31/10/17 | 24/05/18 | 205 |
| 44302 | Bernese Mountain Dog | CLEAR | F | 07/03/14 | 11/06/18 | 1557 |
| 44305 | Coton de Tuléar | CLEAR | F | 03/01/17 | 11/06/18 | 524 |
| 44313 | Bernese Mountain Dog | CLEAR | M | 29/01/17 | 16/06/18 | 503 |
| 44314 | Bernese Mountain Dog | CLEAR | F | 08/01/16 | 16/06/18 | 890 |
| 44329 | Smooth Collie | CLEAR | F | 13/06/17 | 16/06/18 | 368 |
| 44579 | Czechoslovakian wolfdog | CLEAR | F | 28/02/16 | 16/06/18 | 839 |
| 44585 | Czechoslovakian wolfdog | CLEAR | M | 15/11/17 | 15/06/18 | 212 |
| 44680 | Czechoslovakian wolfdog | CLEAR | F | 07/06/16 | 26/06/18 | 749 |
| 44688 | German shepherd | CLEAR | F | 01/03/10 | 25/06/18 | 3038 |
| 44769 | Mixed breed | CLEAR | F | 25/04/14 | 03/07/18 | 1530 |
| 44875 | German shepherd | CLEAR | M | 22/08/11 | 16/07/18 | 2520 |
| 44884 | Czechoslovakian wolfdog | CARRIER | M | 02/06/17 | 16/07/18 | 409 |
| 44897 | Australian shepherd | CLEAR | F | 09/10/17 | 17/07/18 | 281 |
| 44898 | Australian shepherd | CLEAR | F | 09/10/17 | 17/07/18 | 281 |
| 44937 | French Bulldog | CLEAR | F | 07/03/17 | 23/07/18 | 503 |
| 44938 | French Bulldog | CARRIER | M | 27/07/17 | 23/07/18 | 361 |
| 44939 | French Bulldog | CLEAR | M | 04/03/16 | 23/07/18 | 871 |
| 44981 | Bernese Mountain Dog | CLEAR | M | 03/04/17 | 24/07/18 | 477 |
| 45042 | Bernese Mountain Dog | CLEAR | M | 29/12/15 | 01/08/18 | 946 |
| 45140 | Shetland Sheepdog | CARRIER | F | 11/08/15 | 29/08/18 | 1114 |
| 45157 | German shepherd | CARRIER | F | 06/08/11 | 01/09/18 | 2583 |
| 45176 | Czechoslovakian wolfdog | CARRIER | F | 04/12/13 | 03/09/18 | 1734 |

| | | | | | | |
|---|---|---|---|---|---|---|
| 45195 | Shetland Sheepdog | CLEAR | F | 17/12/16 | 05/09/18 | 627 |
| 45334 | Shetland Sheepdog | CLEAR | F | 13/05/18 | 12/09/18 | 122 |
| 45339 | Welsh Corgi Pembroke | AFFECTED | M | 08/06/17 | 16/09/18 | 465 |
| 45340 | Mudi | CLEAR | M | 14/07/15 | 16/09/18 | 1160 |
| 45343 | Mixed breed | CLEAR | F | 10/06/09 | 12/09/18 | 3381 |
| 45386 | Bernese Mountain Dog | CLEAR | M | 10/03/17 | 17/09/18 | 556 |
| 45416 | Welsh Corgi Pembroke | AFFECTED | F | 25/05/16 | 17/09/18 | 845 |
| 45454 | Czechoslovakian wolfdog | CLEAR | F | 14/10/17 | 22/09/18 | 343 |
| 45462 | Barzoi | CARRIER | M | 26/03/18 | 23/09/18 | 181 |
| 45479 | Irish Wolfhound | CLEAR | F | 28/04/17 | 23/09/18 | 513 |
| 45545 | German shepherd | CLEAR | F | 27/10/05 | 14/09/18 | 4705 |
| 45563 | Hovawart | CLEAR | M | 10/02/11 | 24/09/18 | 2783 |
| 45580 | French Bulldog | CLEAR | F | 17/06/18 | 28/08/18 | 72 |
| 45634 | Czechoslovakian wolfdog | CLEAR | M | 03/03/17 | 22/09/18 | 568 |
| 45635 | Czechoslovakian wolfdog | CARRIER | M | 03/03/17 | 22/09/18 | 568 |
| 45654 | Czechoslovakian wolfdog | CARRIER | F | 21/12/16 | 04/10/18 | 652 |
| 45657 | German shepherd | AFFECTED | F | 23/08/08 | 08/10/18 | 3698 |
| 45668 | Shetland Sheepdog | CLEAR | F | 16/06/16 | 08/10/18 | 844 |
| 45726 | Welsh Corgi Pembroke | CARRIER | F | 25/02/17 | 11/10/18 | 593 |
| 45827 | Dobermann Pinscher | CLEAR | M | 21/09/08 | 18/10/18 | 3679 |
| 45828 | Czechoslovakian wolfdog | CARRIER | M | 16/10/17 | 18/10/18 | 367 |
| 45831 | Australian shepherd | CARRIER | F | 15/01/14 | 18/10/18 | 1737 |
| 46050 | German shepherd | CLEAR | M | 29/04/09 | 30/10/18 | 3471 |
| 46152 | Czechoslovakian wolfdog | CARRIER | F | 23/10/16 | 05/11/18 | 743 |
| 46246 | Czechoslovakian wolfdog | CLEAR | F | 19/09/17 | 04/11/18 | 411 |
| 46253 | Rhodesian Ridgeback | CLEAR | F | 05/07/15 | 04/11/18 | 1218 |
| 46283 | White Swiss Shepherd Dog | CLEAR | F | 15/05/17 | 04/11/18 | 538 |
| 46302 | Shetland Sheepdog | CLEAR | M | 28/02/18 | 21/11/18 | 266 |
| 46381 | Welsh Corgi Pembroke | CARRIER | F | 30/01/16 | 28/11/18 | 1033 |
| 46400 | German shepherd | CLEAR | M | 01/08/11 | 29/11/18 | 2677 |
| 46408 | German shepherd | CARRIER | F | 02/01/06 | 30/11/18 | 4715 |
| 46409 | Czechoslovakian wolfdog | CLEAR | F | 10/05/05 | 29/11/18 | 4951 |
| 46486 | Rough Collie | CARRIER | M | 22/02/18 | 05/12/18 | 286 |
| 46488 | Welsh Corgi Pembroke | AFFECTED | M | 08/04/18 | 05/12/18 | 241 |

| | | | | | | |
|---|---|---|---|---|---|---|
| 46489 | Welsh Corgi Pembroke | CARRIER | F | 08/04/18 | 05/12/18 | 241 |
| 46499 | Lapponian Herder | CARRIER | F | 03/10/17 | 10/12/18 | 433 |
| 46664 | German shepherd | CARRIER | M | 03/10/11 | 10/12/18 | 2625 |
| 46677 | German shepherd | CLEAR | F | 24/08/16 | 11/12/18 | 839 |
| 46762 | Australian shepherd | CLEAR | M | 06/06/14 | 19/12/18 | 1657 |
| 46819 | Welsh Corgi Pembroke | AFFECTED | F | 13/03/18 | 03/01/19 | 296 |
| 46863 | Bernese Mountain Dog | CARRIER | M | 20/08/17 | 08/01/19 | 506 |
| 46894 | German shepherd | CARRIER | M | 29/09/14 | 08/01/19 | 1562 |
| 46940 | Czechoslovakian wolfdog | CLEAR | F | 13/10/17 | 05/01/19 | 449 |
| 47037 | French Bulldog | CLEAR | M | 25/04/18 | 10/01/19 | 260 |
| 47134 | Shetland Sheepdog | CLEAR | F | 04/09/18 | 19/01/19 | 137 |
| 47191 | Czechoslovakian wolfdog | CLEAR | | | | |
| 47273 | Rhodesian Ridgeback | CLEAR | F | 07/11/16 | 21/01/19 | 805 |
| 47341 | German shepherd | AFFECTED | M | 01/01/09 | 28/01/19 | 3679 |
| 47354 | Welsh Corgi Pembroke | CARRIER | F | 07/03/17 | 28/01/19 | 692 |
| 47409 | German shepherd | CARRIER | M | 10/06/09 | 30/01/19 | 3521 |
| 47440 | Rough Collie | CLEAR | F | 28/01/18 | 31/01/19 | 368 |
| 47524 | French Bulldog | CARRIER | F | 20/10/17 | 01/02/19 | 469 |
| 47525 | French Bulldog | CLEAR | F | 24/07/17 | 01/02/19 | 557 |
| 47556 | Australian shepherd | CARRIER | F | 23/11/15 | 05/02/19 | 1170 |
| 47587 | White Swiss Shepherd Dog | CARRIER | F | 28/09/14 | 03/02/19 | 1589 |
| 47588 | White Swiss Shepherd Dog | CLEAR | M | 06/09/13 | 03/02/19 | 1976 |
| 47589 | White Swiss Shepherd Dog | CARRIER | F | 21/03/18 | 03/02/19 | 319 |
| 47662 | German shepherd | AFFECTED | M | 10/03/11 | 06/02/19 | 2890 |
| 47731 | German shepherd | CLEAR | F | 20/06/16 | 11/02/19 | 966 |
| 47732 | German shepherd | CLEAR | F | 17/10/09 | 11/02/19 | 3404 |
| 47733 | German shepherd | CLEAR | F | 13/01/13 | 11/02/19 | 2220 |
| 47738 | Chien de Saint-Hubert | CARRIER | M | 11/11/15 | 11/02/19 | 1188 |
| 47741 | Australian shepherd | CLEAR | M | 09/09/18 | 12/02/19 | 156 |
| 47786 | German shepherd | AFFECTED | M | 08/12/08 | 07/02/19 | 3713 |
| 47841 | Bernese Mountain Dog | CLEAR | F | 12/05/16 | 12/02/19 | 1006 |
| 47847 | French Bulldog | CARRIER | M | 21/10/17 | 12/02/19 | 479 |
| 47870 | Hovawart | CLEAR | M | 11/03/17 | 06/02/19 | 697 |
| 47921 | French Bulldog | CLEAR | | | | |

| | | | | | | |
|---|---|---|---|---|---|---|
| 47922 | French Bulldog | CLEAR | | | | |
| 47929 | French Bulldog | CARRIER | M | 11/02/17 | 14/02/19 | 733 |
| 47930 | German shepherd | CLEAR | M | 31/10/18 | 14/02/19 | 106 |
| 47979 | French Bulldog | CLEAR | | | | |
| 48001 | German shepherd | CARRIER | M | 07/02/13 | 19/02/19 | 2203 |
| 48193 | White Swiss Shepherd Dog | CLEAR | F | 08/04/17 | 25/02/19 | 688 |
| 48194 | White Swiss Shepherd Dog | CLEAR | M | 08/04/17 | 25/02/19 | 688 |
| 48220 | Bernese Mountain Dog | CLEAR | M | 04/09/15 | 25/02/19 | 1270 |
| 48221 | Bernese Mountain Dog | CLEAR | F | 12/02/16 | 25/02/19 | 1109 |
| 48226 | German shepherd | CLEAR | F | 13/02/14 | 26/02/19 | 1839 |
| 48227 | French Bulldog | CLEAR | F | 05/08/17 | 26/02/19 | 570 |
| 48256 | French Bulldog | CLEAR | M | 22/09/16 | 26/02/19 | 887 |
| 48315 | Welsh Corgi Pembroke | AFFECTED | F | 05/02/18 | 02/03/19 | 390 |
| 48316 | Boston terrier | CLEAR | F | 06/08/18 | 02/03/19 | 208 |
| 48320 | Boston terrier | CARRIER | F | 28/11/17 | 02/03/19 | 459 |
| 48397 | German shepherd | CLEAR | F | 11/08/17 | 04/03/19 | 570 |
| 48478 | Barzoi | CARRIER | M | 07/11/18 | 10/03/19 | 123 |
| 48532 | French Bulldog | CLEAR | M | 30/09/14 | 05/03/19 | 1617 |
| 48540 | Kerry blue terrier | CLEAR | F | 22/12/15 | 05/03/19 | 1169 |
| 48561 | Australian shepherd | CLEAR | F | 06/12/16 | 07/03/19 | 821 |
| 48562 | Australian shepherd | CLEAR | M | 26/12/16 | 07/03/19 | 801 |
| 48723 | French Bulldog | CARRIER | F | 30/11/17 | 08/03/19 | 463 |
| 48724 | French Bulldog | CLEAR | F | 27/02/18 | 08/03/19 | 374 |
| 48752 | Czechoslovakian wolfdog | CLEAR | F | 02/02/18 | 08/03/19 | 399 |
| 48848 | German shepherd | CLEAR | M | 11/04/14 | 11/03/19 | 1795 |
| 48940 | Australian shepherd | CLEAR | F | 17/05/16 | 11/03/19 | 1028 |
| 48992 | Welsh Corgi Pembroke | AFFECTED | M | 22/02/17 | 20/03/19 | 756 |
| 49132 | German shepherd | CLEAR | F | 10/11/11 | 25/03/19 | 2692 |
| 49364 | Czechoslovakian wolfdog | CLEAR | M | 11/12/17 | 02/04/19 | 477 |
| 49404 | German shepherd | CLEAR | M | 05/07/10 | 03/04/19 | 3194 |
| 49428 | German shepherd | CARRIER | M | 17/05/15 | 05/04/19 | 1419 |
| 49429 | German shepherd | AFFECTED | F | 23/07/17 | 05/04/19 | 621 |
| 49445 | German shepherd | CLEAR | M | 10/01/10 | 04/04/19 | 3371 |
| 49446 | Australian shepherd | CLEAR | F | 17/08/12 | 04/04/19 | 2421 |

| | | | | | | |
|---|---|---|---|---|---|---|
| 49447 | Australian shepherd | CLEAR | M | 29/06/13 | 04/04/19 | 2105 |
| 49448 | Australian shepherd | CLEAR | F | 09/07/15 | 04/04/19 | 1365 |
| 49449 | Australian shepherd | CLEAR | F | 09/07/15 | 04/04/19 | 1365 |
| 49594 | Welsh Corgi Pembroke | CARRIER | M | 11/07/17 | 12/04/19 | 640 |
| 49596 | Canadian Eskimo Dog | CLEAR | M | 28/04/17 | 12/04/19 | 714 |
| 49717 | Shetland shepherd | CLEAR | F | 15/04/18 | 17/04/19 | 367 |
| 49718 | Giant poodle | CLEAR | F | 25/07/17 | 17/04/19 | 631 |
| 49751 | Australian shepherd | CLEAR | F | 31/12/18 | 17/04/19 | 107 |
| 49832 | Czechoslovakian wolfdog | CLEAR | F | 15/03/14 | 06/05/19 | 1878 |
| 50082 | Czechoslovakian wolfdog | CARRIER | F | 21/11/18 | 06/05/19 | 166 |
| 50083 | Czechoslovakian wolfdog | CLEAR | F | 21/11/18 | 06/05/19 | 166 |
| 50254 | Hovawart | CLEAR | M | 03/11/17 | 30/04/19 | 543 |
| 50280 | French Bulldog | CLEAR | | | | |
| 50375 | Chien de Saint-Hubert | CLEAR | M | 24/01/17 | 13/05/19 | 839 |
| 50376 | Chien de Saint-Hubert | CARRIER | F | 18/01/19 | 13/05/19 | 115 |
| 50499 | German shepherd | CARRIER | | | | |
| 50651 | Kerry blue terrier | CLEAR | | | | |
| 50773 | Bernese Mountain Dog | CARRIER | F | 08/11/16 | 27/05/19 | 930 |
| 50841 | Australian shepherd | CLEAR | M | 04/03/18 | 28/05/19 | 450 |
| 50888 | Brussels Griffon | CLEAR | | | | |
| 50991 | White Swiss Shepherd Dog | CARRIER | | | | |
| 50995 | Czechoslovakian wolfdog | CLEAR | M | 11/05/17 | 05/06/19 | 755 |
| 51210 | Czechoslovakian wolfdog | CLEAR | M | 28/03/16 | 12/06/19 | 1171 |
| 51236 | White Swiss Shepherd Dog | CLEAR | | | | |
| 51310 | Barzoi | CARRIER | F | 08/08/11 | 17/06/19 | 2870 |
| 51354 | Welsh Corgi Pembroke | CARRIER | M | 14/05/19 | 13/06/19 | 30 |
| 51355 | Welsh Corgi Pembroke | AFFECTED | M | 14/05/19 | 13/06/19 | 30 |
| 51356 | Welsh Corgi Pembroke | CARRIER | F | 14/05/19 | 13/06/19 | 30 |
| 51416 | German shepherd | CLEAR | F | 25/04/08 | 17/06/19 | 4070 |
| 51435 | German shepherd | CLEAR | M | 02/10/18 | 18/06/19 | 259 |
| 51437 | Czechoslovakian wolfdog | CLEAR | M | 31/01/18 | 20/06/19 | 505 |
| 51465 | Mixed breed | CLEAR | F | 09/06/10 | 21/06/19 | 3299 |
| 51503 | White Swiss Shepherd Dog | CLEAR | F | 11/01/17 | 13/06/19 | 883 |
| 51572 | Boston terrier | CLEAR | F | 23/07/17 | 24/06/19 | 701 |

| | | | | | | |
|---|---|---|---|---|---|---|
| 51573 | Boston terrier | CARRIER | M | 23/07/17 | 24/06/19 | 701 |
| 51633 | German shepherd | AFFECTED | M | 03/11/09 | 26/06/19 | 3522 |
| 51794 | Mixed breed | CARRIER | M | 17/09/03 | 08/07/19 | 5773 |
| 51835 | French Bulldog | CLEAR | | | | |
| 51885 | Bernese Mountain Dog | CLEAR | F | 11/03/16 | 09/07/19 | 1215 |
| 51936 | Boston terrier | CLEAR | | | | |
| 51978 | Hovawart | CARRIER | F | 30/03/18 | 07/07/19 | 464 |
| 52152 | Czechoslovakian wolfdog | CLEAR | F | 06/12/17 | 28/07/19 | 599 |
| 52179 | White Swiss Shepherd Dog | CLEAR | | | | |
| 52274 | German shepherd | CARRIER | M | 25/08/08 | 29/07/19 | 3990 |
| 52292 | German shepherd | CLEAR | M | 16/04/17 | 30/07/19 | 835 |
| 52398 | French Bulldog | CARRIER | F | 10/11/15 | 17/07/19 | 1345 |
| 52399 | Bernese Mountain Dog | CLEAR | M | 20/12/15 | 22/07/19 | 1310 |
| 52410 | Poodle | CLEAR | M | 10/08/14 | 17/07/19 | 1802 |
| 52560 | Belgian shepherd Malinois | CLEAR | F | 20/01/17 | 29/08/19 | 951 |
| 52588 | Mixed breed | CLEAR | F | 01/05/13 | 30/08/19 | 2312 |
| 52631 | German shepherd | CARRIER | F | 22/09/13 | 23/08/19 | 2161 |
| 52632 | German shepherd | CLEAR | M | 22/01/15 | 23/08/19 | 1674 |
| 52909 | Welsh Corgi Pembroke | CARRIER | M | 19/10/18 | 09/09/19 | 325 |
| 52974 | French Bulldog | CLEAR | F | 29/08/16 | 04/09/19 | 1101 |
| 52983 | Australian shepherd | CARRIER | F | 25/07/16 | | |
| 53053 | Rough Collie | CLEAR | | | | |
| 53061 | Poodle | CLEAR | | | | |
| 53062 | Czechoslovakian wolfdog | CLEAR | M | 30/10/18 | 15/09/19 | 320 |
| 53120 | White Swiss Shepherd Dog | CLEAR | | | | |
| 53170 | Bernese Mountain Dog | CLEAR | M | 09/05/18 | 26/08/19 | 474 |
| 53291 | Czechoslovakian wolfdog | CLEAR | | 14/12/17 | 19/09/19 | 644 |
| 53307 | Bernese Mountain Dog | CARRIER | F | 03/04/18 | 24/07/19 | 477 |
| 53318 | Czechoslovakian wolfdog | CLEAR | F | 16/10/16 | 20/09/19 | 1069 |
| 53325 | Miniature poodle | CLEAR | M | 05/03/17 | 23/09/19 | 932 |
| 53334 | Australian shepherd | CLEAR | | | | |
| 53374 | White Swiss Shepherd Dog | CLEAR | M | 12/11/17 | 23/09/19 | 680 |
| 53375 | White Swiss Shepherd Dog | CLEAR | F | 30/06/16 | 23/09/19 | 1180 |
| 53386 | White Swiss Shepherd Dog | CLEAR | | | | |

| | | | | | | |
|---|---|---|---|---|---|---|
| 53390 | Australian shepherd | CLEAR | F | 08/12/15 | 24/09/19 | 1386 |
| 53411 | Riesenschnauzer | CLEAR | | | | |
| 53414 | Smooth collie | CARRIER | | | | |
| 53417 | Welsh Corgi Pembroke | CLEAR | | 21/01/19 | 29/09/19 | 251 |
| 53533 | German shepherd | CLEAR | F | 31/12/12 | 30/09/19 | 2464 |
| 53543 | French Bulldog | AFFECTED | F | 24/03/19 | 24/09/19 | 184 |
| 53558 | German shepherd | CLEAR | F | 01/07/19 | 01/10/19 | 92 |
| 53566 | Czechoslovakian wolfdog | CLEAR | F | 26/12/16 | 01/10/19 | 1009 |
| 53617 | Beagle | CLEAR | M | 12/05/14 | 02/10/19 | 1969 |
| 53642 | White Swiss Shepherd Dog | CLEAR | | | | |
| 53734 | White Swiss Shepherd Dog | CLEAR | | | | |
| 53741 | Czechoslovakian wolfdog | CARRIER | F | 19/01/12 | 09/10/19 | 2820 |
| 53745 | Czechoslovakian wolfdog | CLEAR | | | | |
| 53805 | Czechoslovakian wolfdog | CLEAR | | | | |
| 53836 | Mixed breed | CLEAR | F | 01/02/09 | 02/10/19 | 3895 |
| 53845 | Miniature poodle | CLEAR | | | | |
| 53860 | French Bulldog | CARRIER | | 18/12/16 | 09/10/19 | 1025 |
| 53903 | German shepherd | AFFECTED | F | 14/03/11 | 15/10/19 | 3137 |
| 53915 | French Bulldog | CLEAR | F | 13/12/18 | 16/10/19 | 307 |
| 53916 | French Bulldog | CLEAR | F | 18/03/19 | 16/10/19 | 212 |
| 53917 | French Bulldog | CLEAR | M | 22/12/18 | 16/10/19 | 298 |
| 53918 | French Bulldog | CARRIER | F | 02/12/18 | 16/10/19 | 318 |
| 53966 | Hovawart | CARRIER | M | 22/03/17 | 17/10/19 | 939 |
| 53969 | Australian shepherd | CLEAR | M | 10/02/10 | 15/10/19 | 3534 |
| 53996 | Bernese Mountain Dog | CARRIER | M | 19/04/12 | 18/10/19 | 2738 |
| 53997 | Miniature poodle | CLEAR | F | 18/02/17 | 20/10/19 | 974 |
| 54007 | Rhodesian Ridgeback | CLEAR | M | 29/08/16 | 22/10/19 | 1149 |
| 54047 | Bernese Mountain Dog | CARRIER | | | | |
| 54128 | Bernese Mountain Dog | CARRIER | F | 27/12/17 | 25/10/19 | 667 |
| 54218 | Shetland shepherd | CLEAR | M | 13/10/18 | 01/11/19 | 384 |
| 54508 | Giant poodle | CLEAR | M | 04/04/11 | 07/11/19 | 3139 |
| 54509 | Shetland shepherd | CLEAR | M | 05/08/19 | 07/11/19 | 94 |
| 54547 | German shepherd | CLEAR | | | | |
| 54563 | Czechoslovakian wolfdog | CLEAR | M | 15/11/17 | 11/11/19 | 726 |

| | | | | | | |
|---|---|---|---|---|---|---|
| 54566 | Australian shepherd | CLEAR | F | 29/08/19 | 14/11/19 | 77 |
| 54602 | Australian shepherd | CLEAR | M | 22/11/16 | 15/11/19 | 1088 |
| 54603 | Australian shepherd | CLEAR | F | 18/09/17 | 15/11/19 | 788 |
| 54633 | Czechoslovakian wolfdog | CARRIER | | | | |
| 54765 | German shepherd | AFFECTED | M | 01/06/14 | 25/11/19 | 2003 |
| 54806 | Belgian shepherd Malinois | CLEAR | F | 04/04/15 | 22/11/19 | 1693 |
| 54807 | Dutch Shepherd Dog | CLEAR | F | 04/04/15 | 22/11/19 | 1693 |
| 54808 | Belgian shepherd Malinois | CLEAR | F | 12/06/14 | 22/11/19 | 1989 |
| 54809 | Belgian shepherd Malinois | CLEAR | | | | |
| 54810 | Belgian shepherd Malinois | CLEAR | M | 13/04/18 | 22/11/19 | 588 |
| 54817 | Czechoslovakian wolfdog | CLEAR | | | | |
| 54823 | Czechoslovakian wolfdog | CLEAR | | | | |
| 54827 | Czechoslovakian wolfdog | CLEAR | | | | |
| 54841 | White Swiss Shepherd Dog | CLEAR | | | | |
| 54847 | Bernese Mountain Dog | CLEAR | | | | |
| 54909 | Rhodesian Ridgeback | CLEAR | | | | |
| 54913 | German shepherd | CLEAR | M | 31/10/15 | 02/12/19 | 1493 |
| 54914 | German shepherd | CLEAR | F | 14/11/17 | 03/12/19 | 749 |
| 54944 | Chien de Saint-Hubert | CARRIER | | | | |
| 55018 | Belgian shepherd Tervueren | CLEAR | | | | |
| 55060 | German shepherd | CARRIER | F | 24/11/17 | 07/12/19 | 743 |
| 55064 | German shepherd | CLEAR | M | 01/12/09 | 02/12/19 | 3653 |
| 55192 | Chien de Saint-Hubert | AFFECTED | | | | |
| 55216 | Weimaraner | CLEAR | | | | |
| 55233 | Border collie | CLEAR | | | | |
| 55382 | German shepherd | | M | 06/03/12 | 05/12/19 | 2830 |
| 55429 | German shepherd | | M | 25/01/19 | 17/12/19 | 326 |
| 55565 | Czechoslovakian wolfdog | CLEAR | F | 30/09/16 | 02/01/20 | 1189 |
| 55607 | German shepherd | CARRIER | M | 25/01/19 | 08/01/20 | 348 |
| 55625 | Czechoslovakian wolfdog | CLEAR | F | 09/11/17 | 08/01/20 | 790 |
| 55662 | French Bulldog | CLEAR | | | | |
| 55715 | Mixed breed | CLEAR | M | 02/01/10 | 05/07/19 | 3471 |
| 55746 | Czechoslovakian wolfdog | CLEAR | F | 21/12/16 | 13/01/20 | 1118 |
| 55752 | Rhodesian Ridgeback | CLEAR | F | 24/06/08 | 04/12/19 | 4180 |

| | | | | | | |
|---|---|---|---|---|---|---|
| 55779 | Bernese Mountain Dog | CLEAR | M | 01/03/16 | 15/01/20 | 1415 |
| 55779 | Bernese Mountain Dog | CLEAR | M | 01/03/16 | 15/01/20 | 1415 |
| 55839 | Boston terrier | CLEAR | | | | |
| 55840 | Boston terrier | CLEAR | | | | |
| 55851 | Miniature poodle | CLEAR | | | | |
| 55858 | Rhodesian Ridgeback | CARRIER | | | | |
| 55859 | Welsh Corgi Pembroke | AFFECTED | | | | |
| 55862 | Weimaraner | CLEAR | | | | |
| 55864 | Australian shepherd | CLEAR | | | | |
| 55933 | Australian shepherd | CLEAR | M | 31/07/17 | 17/01/20 | 900 |
| 55956 | White Swiss Shepherd Dog | CARRIER | | | | |
| 56056 | German shepherd | CLEAR | M | 30/07/13 | 23/01/20 | 2368 |
| 56062 | Czechoslovakian wolfdog | AFFECTED | F | 02/11/14 | 22/01/20 | 1907 |
| 56133 | Flat Coated Retriever | CARRIER | M | 09/06/09 | 28/01/20 | 3885 |
| 56146 | German shepherd | CLEAR | M | 16/12/18 | 27/01/20 | 407 |
| 56275 | French Bulldog | CLEAR | | | | |
| 56368 | Miniature poodle | CLEAR | F | 17/03/16 | 07/02/20 | 1422 |
| 56381 | Barzoi | | F | 18/10/19 | 07/02/20 | 112 |
| 56384 | American Staffordshire Terrier | | F | 08/12/18 | 06/02/20 | 425 |
| 56387 | German shepherd | | M | 15/02/14 | 11/02/20 | 2187 |
| 56397 | Australian shepherd | CLEAR | | | | |
| 56465 | Czechoslovakian wolfdog | CARRIER | | | | |
| 56467 | Belgian shepherd Tervueren | CLEAR | | | | |
| 56519 | Kerry blue terrier | CLEAR | | | | |
| 56581 | Welsh Corgi Pembroke | CARRIER | F | 06/12/19 | 17/02/20 | 73 |
| 56588 | German shepherd | CARRIER | F | 10/02/19 | 15/02/20 | 370 |
| 56630 | Hovawart | CARRIER | M | 14/11/19 | 15/02/20 | 93 |
| 56644 | Hovawart | CLEAR | F | 08/06/16 | 13/02/20 | 1345 |
| 56645 | Hovawart | CLEAR | F | 16/12/19 | 13/02/20 | 59 |
| 56646 | Hovawart | CARRIER | F | 30/11/17 | 13/02/20 | 805 |
| 56681 | Hovawart | CARRIER | | | | |
| 56697 | Welsh Corgi Pembroke | AFFECTED | | | | |
| 56759 | Weimaraner | CLEAR | | | | |
| 56907 | German shepherd | AFFECTED | M | 17/02/11 | 09/03/20 | 3308 |

| | | | | | | | |
|---|---|---|---|---|---|---|---|
| 56908 | French Bulldog | CLEAR | | | | |
| 57044 | Labrador retriever | CLEAR | F | 01/06/11 | 15/04/20 | 3241 |
| 57182 | Welsh Corgi Pembroke | CARRIER | M | 21/02/20 | 16/04/20 | 55 |
| 57183 | Welsh Corgi Pembroke | CLEAR | M | 21/02/20 | 16/04/20 | 55 |
| 57184 | Welsh Corgi Pembroke | CLEAR | F | 21/02/20 | 16/04/20 | 55 |
| 57269 | Hovawart | CLEAR | F | 26/04/17 | 07/05/20 | 1107 |
| 57273 | White Swiss Shepherd Dog | CLEAR | M | 18/02/20 | 04/05/20 | 76 |
| 57274 | White Swiss Shepherd Dog | CARRIER | M | 18/02/20 | 04/05/20 | 76 |
| 57277 | Hovawart | CLEAR | M | 05/02/18 | 07/05/20 | 822 |
| 57301 | French Bulldog | CLEAR | M | 06/05/19 | 07/05/20 | 367 |
| 57304 | Czechoslovakian wolfdog | CLEAR | M | 10/04/14 | 06/05/20 | 2218 |
| 57363 | Italian rough-hair segugio | AFFECTED | M | 16/04/08 | 08/05/20 | 4405 |
| 57388 | Staffordshire bull terrier | CLEAR | | | | |
| 57418 | Miniature poodle | CLEAR | | | | |
| 57419 | Miniature poodle | CLEAR | | | | |
| 57424 | Pug | CLEAR | | | | |
| 57513 | Czechoslovakian wolfdog | CLEAR | | | | |
| 57517 | Czechoslovakian wolfdog | CLEAR | | | | |
| 57550 | White Swiss Shepherd Dog | CARRIER | M | 30/06/16 | 18/05/20 | 1418 |
| 57551 | White Swiss Shepherd Dog | CARRIER | M | 09/03/19 | 18/05/20 | 436 |
| 57575 | French Bulldog | CARRIER | F | 10/11/15 | 20/05/20 | 1653 |
| 57576 | French Bulldog | CLEAR | M | 15/12/16 | 20/05/20 | 1252 |
| 57598 | German shepherd | CARRIER | M | 01/12/11 | 20/05/20 | 3093 |
| 57604 | French Bulldog | CARRIER | F | | | |
| 57605 | French Bulldog | CLEAR | F | | | |
| 57639 | Czechoslovakian wolfdog | CLEAR | F | 03/11/17 | 26/05/20 | 935 |
| 57662 | French Bulldog | CLEAR | M | 01/02/19 | 25/05/20 | 479 |
| 57683 | German shepherd | CLEAR | F | 14/07/11 | 27/05/20 | 3240 |
| 57689 | German shepherd | CLEAR | M | 19/03/19 | 19/05/20 | 427 |
| 57721 | Shetland shepherd | CLEAR | F | 05/12/16 | 28/05/20 | 1270 |
| 57889 | German shepherd | CLEAR | | | | |
| 57904 | French Bulldog | CLEAR | | | | |
| 57915 | Hovawart | CLEAR | M | 29/05/19 | 08/06/20 | 376 |
| 57940 | Australian shepherd | CLEAR | M | 30/01/12 | 03/06/20 | 3047 |

| | | | | | | |
|---|---|---|---|---|---|---|
| 57941 | Rottweiler | CLEAR | F | 21/05/16 | 03/06/20 | 1474 |
| 57992 | German shepherd | AFFECTED | M | 30/04/12 | 03/06/20 | 2956 |
| 58036 | German shepherd | CLEAR | | | | |
| 58039 | Toy poodle | CARRIER | F | 15/09/17 | 16/06/20 | 1005 |
| 58040 | Toy poodle | CLEAR | M | 08/08/17 | 16/06/20 | 1043 |
| 58041 | Australian shepherd | CLEAR | F | 23/12/13 | 17/06/20 | 2368 |
| 58056 | French Bulldog | CLEAR | F | 10/06/19 | 15/06/20 | 371 |
| 58062 | German shepherd | AFFECTED | M | 15/01/11 | 16/06/20 | 3440 |
| 58073 | Bernese Mountain Dog | CLEAR | M | 07/12/14 | 17/06/20 | 2019 |
| 58094 | Australian shepherd | CLEAR | M | 25/08/17 | 15/06/20 | 1025 |
| 58097 | Poodle | CLEAR | F | 11/11/19 | 16/06/20 | 218 |
| 58113 | French Bulldog | CLEAR | F | 29/11/17 | 15/06/20 | 929 |
| 58114 | French Bulldog | CARRIER | M | 27/04/19 | 15/06/20 | 415 |
| 58115 | French Bulldog | CLEAR | F | 29/11/17 | 15/06/20 | 929 |
| 58123 | French Bulldog | CLEAR | M | 05/05/18 | 22/06/20 | 779 |
| 58143 | German shepherd | CLEAR | F | 11/11/13 | 23/06/20 | 2416 |
| 58170 | Czechoslovakian wolfdog | CLEAR | M | 10/04/14 | 23/06/20 | 2266 |
| 58197 | Kerry blue terrier | CLEAR | | | | |
| 58236 | French Bulldog | CLEAR | F | 21/06/18 | 24/06/20 | 734 |
| 58237 | French Bulldog | CLEAR | F | 27/04/19 | 24/06/20 | 424 |
| 58248 | Cavalier King Charles Spaniel | CARRIER | M | 30/03/18 | 29/06/20 | 822 |
| 58249 | Cavalier King Charles Spaniel | AFFECTED | M | 25/04/17 | 29/06/20 | 1161 |
| 58250 | Cavalier King Charles Spaniel | AFFECTED | F | 03/01/18 | 29/06/20 | 908 |
| 58251 | Czechoslovakian wolfdog | CLEAR | F | 24/05/16 | 01/07/20 | 1499 |
| 58262 | Bernese Mountain Dog | CLEAR | | | | |
| 58263 | Bernese Mountain Dog | CLEAR | | | | |
| 58264 | Bernese Mountain Dog | CARRIER | | | | |
| 58265 | Bernese Mountain Dog | CLEAR | | | | |
| 58266 | Bernese Mountain Dog | CLEAR | | | | |
| 58267 | Bernese Mountain Dog | CLEAR | | | | |
| 58268 | Bernese Mountain Dog | CLEAR | | | | |
| 58269 | Bernese Mountain Dog | CLEAR | | | | |
| 58270 | Bernese Mountain Dog | CLEAR | | | | |
| 58285 | Australian shepherd | CLEAR | F | 30/12/19 | 27/06/20 | 180 |

| | | | | | | |
|---|---|---|---|---|---|---|
| 58448 | Czechoslovakian wolfdog | CLEAR | F | 11/11/18 | 09/07/20 | 606 |
| 58470 | Miniature poodle | CLEAR | F | 13/04/19 | 12/07/20 | 456 |
| 58545 | Czechoslovakian wolfdog | CARRIER | M | 27/12/17 | 15/07/20 | 931 |
| 58628 | French Bulldog | CLEAR | F | 14/06/16 | 16/07/20 | 1493 |
| 58662 | Czechoslovakian wolfdog | CLEAR | M | 16/02/20 | 21/07/20 | 156 |
| 58664 | Czechoslovakian wolfdog | CARRIER | M | 16/12/18 | 21/07/20 | 583 |
| 58674 | French Bulldog | AFFECTED | F | 27/12/18 | 21/07/20 | 572 |
| 58759 | German shepherd | CLEAR | | | | |
| 58762 | German shepherd | CLEAR | M | 26/04/16 | 24/07/20 | 1550 |
| 58793 | Australian shepherd | CLEAR | F | 13/03/16 | 27/07/20 | 1597 |
| 58795 | German shepherd | CLEAR | F | 01/05/12 | 28/07/20 | 3010 |
| 58842 | Rough Collie | CLEAR | F | 29/02/16 | 29/07/20 | 1612 |
| 58843 | Rough Collie | CARRIER | F | 15/02/16 | 29/07/20 | 1626 |
| 58874 | White Swiss Shepherd Dog | CLEAR | F | 13/02/17 | 30/07/20 | 1263 |
| 58895 | French Bulldog | CLEAR | | | | |
| 58911 | Australian shepherd | CLEAR | M | 20/04/18 | 31/07/20 | 833 |
| 58921 | Australian shepherd | CARRIER | F | 18/10/15 | 04/08/20 | 1752 |
| 58992 | German shepherd | AFFECTED | M | 10/08/10 | 05/08/20 | 3648 |
| 59056 | Czechoslovakian wolfdog | CARRIER | F | 10/11/07 | 12/08/20 | 4659 |
| 59057 | Rottweiler | CLEAR | M | 18/08/11 | 12/08/20 | 3282 |
| 59063 | German shepherd | AFFECTED | F | 28/08/09 | 14/08/20 | 4004 |
| 59065 | German shepherd | CLEAR | F | 03/08/09 | 04/08/20 | 4019 |
| 59099 | French Bulldog | CLEAR | | | | |
| 59100 | French Bulldog | CLEAR | | | | |
| 59139 | Bernese Mountain Dog | CLEAR | F | 01/02/18 | 24/08/20 | 935 |
| 59160 | French Bulldog | CARRIER | F | 12/04/17 | 24/08/20 | 1230 |
| 59161 | French Bulldog | AFFECTED | F | 26/02/17 | 24/08/20 | 1275 |
| 59162 | French Bulldog | CLEAR | M | 07/03/19 | 24/08/20 | 536 |
| 59218 | Czechoslovakian wolfdog | CLEAR | M | 02/07/15 | 28/05/20 | 1792 |
| 59251 | Czechoslovakian wolfdog | CLEAR | M | 15/04/20 | 28/08/20 | 135 |
| 59263 | Rough Collie | CARRIER | F | 25/02/20 | 31/08/20 | 188 |
| 59264 | Rough Collie | CARRIER | F | 12/03/20 | 31/08/20 | 172 |
| 59298 | Australian shepherd | CLEAR | F | 03/09/18 | 01/09/20 | 729 |
| 59328 | Australian shepherd | CLEAR | | | | |

| | | | | | | | |
|---|---|---|---|---|---|---|---|
| 59354 | Bernese Mountain Dog | CLEAR | | | | |
| 59377 | Bernese Mountain Dog | CLEAR | M | 20/01/16 | 02/09/20 | 1687 |
| 59385 | Chien de Saint-Hubert | CARRIER | F | 12/11/17 | 02/09/20 | 1025 |
| 59389 | French Bulldog | CARRIER | | | | |
| 59390 | French Bulldog | CLEAR | | | | |
| 59391 | German shepherd | CLEAR | F | 08/08/17 | 07/09/20 | 1126 |
| 59402 | Toy poodle | CLEAR | F | 14/07/20 | 07/09/20 | 55 |
| 59403 | Toy poodle | CLEAR | M | 14/07/20 | 07/09/20 | 55 |
| 59404 | Toy poodle | CARRIER | M | 14/07/20 | 07/09/20 | 55 |
| 59405 | Toy poodle | CARRIER | F | 14/07/20 | 07/09/20 | 55 |
| 59438 | German shepherd | AFFECTED | F | 03/03/08 | 07/09/20 | 4571 |
| 59532 | Bernese Mountain Dog | CLEAR | F | 13/09/17 | 10/09/20 | 1093 |
| 59581 | French Bulldog | CARRIER | F | 02/07/19 | 17/09/20 | 443 |
| 59590 | Australian shepherd | CLEAR | F | 12/04/15 | 14/09/20 | 1982 |
| 59597 | Rottweiler | CLEAR | M | 31/03/19 | 11/08/20 | 499 |
| 59635 | American akita | CLEAR | M | 14/11/14 | 15/09/20 | 2132 |
| 59640 | German shepherd | CLEAR | | | | |
| 59655 | Bernese Mountain Dog | CARRIER | F | 04/10/13 | 12/09/20 | 2535 |
| 59699 | Czechoslovakian wolfdog | CLEAR | M | 04/06/19 | 21/09/20 | 475 |
| 59706 | Toy poodle | CLEAR | F | 09/09/18 | 21/09/20 | 743 |
| 59807 | French Bulldog | AFFECTED | F | 10/08/20 | 22/09/20 | 43 |
| 59808 | French Bulldog | AFFECTED | F | 10/08/20 | 22/09/20 | 43 |
| 59812 | Czechoslovakian wolfdog | CLEAR | F | 23/01/19 | 29/09/20 | 615 |
| 59845 | Bernese Mountain Dog | CARRIER | F | 16/02/20 | 29/09/20 | 226 |
| 59985 | Tibetan spaniel | CLEAR | | | | |
| 59996 | German shepherd | CARRIER | M | 29/09/17 | 25/08/20 | 1061 |
| 60016 | Welsh Corgi Pembroke | CLEAR | M | 14/09/19 | 30/09/20 | 382 |
| 60017 | Welsh Corgi Pembroke | CARRIER | F | 14/09/19 | 30/09/20 | 382 |
| 60018 | Welsh Corgi Pembroke | CLEAR | F | 14/09/19 | 30/09/20 | 382 |
| 60019 | Welsh Corgi Pembroke | AFFECTED | | | | |
| 60020 | Welsh Corgi Pembroke | CARRIER | M | 01/02/15 | 30/09/20 | 2068 |
| 60021 | Welsh Corgi Pembroke | CARRIER | F | 01/06/17 | 30/09/20 | 1217 |
| 60112 | German shepherd | CLEAR | M | 12/09/16 | 09/10/20 | 1488 |
| 60190 | French Bulldog | CARRIER | F | 10/10/17 | 16/10/20 | 1102 |

| | | | | | | |
|---|---|---|---|---|---|---|
| 60193 | French Bulldog | CARRIER | F | 02/01/19 | 03/10/20 | 640 |
| 60255 | French Bulldog | CARRIER | F | 01/05/18 | 13/10/20 | 896 |
| 60268 | French Bulldog | AFFECTED | | | | |
| 60271 | Australian shepherd | CARRIER | F | 02/10/16 | 17/10/20 | 1476 |
| 60341 | Czechoslovakian wolfdog | CLEAR | M | 02/03/19 | 19/10/20 | 597 |
| 60395 | Poodle | CLEAR | M | 28/09/19 | 23/10/20 | 391 |
| 60403 | Czechoslovakian wolfdog | CLEAR | M | 14/10/19 | 23/10/20 | 375 |
| 60465 | French Bulldog | CLEAR | F | 31/10/19 | 27/10/20 | 362 |
| 60470 | Czechoslovakian wolfdog | CLEAR | F | 15/09/19 | 23/10/20 | 404 |
| 60535 | German shepherd | CLEAR | M | 17/03/16 | 29/10/20 | 1687 |
| 60564 | Welsh Corgi Pembroke | CARRIER | F | 24/04/17 | 28/10/20 | 1283 |
| 60627 | Rhodesian Ridgeback | CLEAR | F | 27/06/18 | 15/10/20 | 841 |
| 60637 | Bernese Mountain Dog | CLEAR | F | 30/07/19 | 03/11/20 | 462 |
| 60690 | German shepherd | CLEAR | F | 20/07/19 | 09/11/20 | 478 |
| 60818 | Welsh Corgi Pembroke | CARRIER | | | | |
| 60832 | Shetland shepherd | CLEAR | M | 05/08/19 | 13/11/20 | 466 |
| 60850 | Welsh Corgi Pembroke | CARRIER | | | | |
| 60859 | French Bulldog | CLEAR | | | | |
| 60890 | French Bulldog | CARRIER | F | 29/05/19 | 13/11/20 | 534 |
| 60897 | French Bulldog | CLEAR | M | 02/10/19 | 09/11/20 | 404 |
| 60919 | French Bulldog | CLEAR | F | 30/01/15 | 18/11/20 | 2119 |
| 60933 | Australian shepherd | CLEAR | M | 28/04/20 | 17/11/20 | 203 |
| 60938 | White Swiss Shepherd Dog | CLEAR | | | | |
| 60952 | Czechoslovakian wolfdog | CLEAR | | 03/02/19 | 13/11/20 | 649 |
| 60955 | French Bulldog | CARRIER | F | 10/08/20 | 20/11/20 | 102 |
| 60962 | White Swiss Shepherd Dog | CLEAR | | | | |
| 61032 | Poodle | CLEAR | F | 13/11/16 | 24/11/20 | 1472 |
| 61043 | French Bulldog | CLEAR | F | 06/05/19 | 26/11/20 | 570 |
| 61054 | French Bulldog | CLEAR | F | 31/07/17 | 24/06/19 | 693 |
| 61060 | Czechoslovakian wolfdog | CLEAR | | | | |
| 61089 | Czechoslovakian wolfdog | CARRIER | M | 28/02/19 | 01/12/20 | 642 |
| 61091 | Poodle | CARRIER | F | 15/07/19 | 01/12/20 | 505 |
| 61096 | German shepherd | CLEAR | | | | |
| 61096 | German shepherd | CARRIER | | | | |

| | | | | | | |
|---|---|---|---|---|---|---|
| 61102 | Czechoslovakian wolfdog | CLEAR | M | 11/10/19 | 30/11/20 | 416 |
| 61368 | Czechoslovakian wolfdog | CLEAR | F | 15/04/19 | 26/11/20 | 591 |
| 61461 | Bernese Mountain Dog | CLEAR | F | 21/12/16 | 15/12/20 | 1455 |
| 61538 | French Bulldog | CARRIER | M | 09/09/20 | 11/12/20 | 93 |
| 61539 | French Bulldog | CLEAR | F | 10/01/18 | 11/12/20 | 1066 |
| 61587 | Toy poodle | CLEAR | F | 25/12/19 | 21/12/20 | 362 |
| 61588 | Toy poodle | CLEAR | M | 25/08/19 | 21/12/20 | 484 |
| 61594 | German shepherd | CARRIER | F | 10/02/19 | 31/12/20 | 690 |
| 61610 | White Swiss Shepherd Dog | CLEAR | | | | |
| 61657 | Poodle | CARRIER | M | 02/09/19 | 30/12/20 | 485 |
| 61664 | Czechoslovakian wolfdog | CLEAR | F | 07/02/20 | 07/01/21 | 335 |
| 61689 | French Bulldog | CLEAR | F | 09/07/18 | 05/01/21 | 911 |
| 61690 | French Bulldog | CLEAR | M | 01/05/16 | 05/01/21 | 1710 |
| 61702 | White Swiss Shepherd Dog | CLEAR | | | | |
| 61726 | Border collie | CLEAR | F | 19/02/19 | 07/01/21 | 688 |
| 61814 | French Bulldog | CLEAR | F | 12/03/20 | 15/01/21 | 309 |
| 61818 | Czechoslovakian wolfdog | CLEAR | F | 17/03/17 | 13/01/21 | 1398 |
| 61822 | French Bulldog | CLEAR | M | 06/05/12 | 13/01/21 | 3174 |
| 61825 | Bernese Mountain Dog | CLEAR | | | | |
| 61831 | German shepherd | CLEAR | F | 24/04/15 | 13/01/21 | 2091 |
| 61832 | German shepherd | CARRIER | F | 27/03/14 | 13/01/21 | 2484 |
| 61872 | French Bulldog | CARRIER | F | 12/05/19 | 18/01/21 | 617 |
| 61903 | Australian shepherd | CLEAR | | | | |
| 61904 | Australian shepherd | CLEAR | | | | |
| 61905 | Bernese Mountain Dog | CARRIER | | | | |
| 61906 | Czechoslovakian wolfdog | CARRIER | M | 01/10/19 | 02/01/21 | 459 |
| 61916 | Shetland shepherd | CLEAR | F | 11/10/19 | 18/01/21 | 465 |
| 61927 | French Bulldog | AFFECTED | F | 05/11/19 | 19/01/21 | 441 |
| 61928 | French Bulldog | CLEAR | M | 04/01/19 | 19/01/21 | 746 |
| 62016 | Belgian shepherd Malinois | CLEAR | | | | |
| 62047 | Australian shepherd | CLEAR | F | 24/04/17 | 20/01/21 | 1367 |
| 62075 | German shepherd | CLEAR | M | 16/05/11 | 21/01/21 | 3538 |
| 62089 | Czechoslovakian wolfdog | CARRIER | F | 24/12/18 | 26/01/21 | 764 |
| 62099 | White Swiss Shepherd Dog | CLEAR | | | | |

| | | | | | | |
|---|---|---|---|---|---|---|
| 62133 | Australian shepherd | CLEAR | M | 12/05/17 | 26/01/21 | 1355 |
| 62141 | Boxer | AFFECTED | M | 01/07/00 | 27/01/21 | 7515 |
| 62159 | Toy poodle | CLEAR | M | 30/11/19 | 15/12/20 | 381 |
| 62206 | German shepherd | CLEAR | F | 28/07/18 | 01/02/21 | 919 |
| 62230 | French Bulldog | CLEAR | | | | |
| 62263 | German shepherd | CLEAR | M | 20/01/12 | 02/02/21 | 3301 |
| 62279 | Czechoslovakian wolfdog | CLEAR | M | 15/09/19 | 27/01/21 | 500 |
| 62285 | Czechoslovakian wolfdog | CLEAR | M | 16/12/19 | 26/01/21 | 407 |
| 62289 | German shepherd | CLEAR | F | 30/11/08 | 02/02/21 | 4447 |
| 62297 | German shepherd | CLEAR | F | 30/10/20 | 01/02/21 | 94 |
| 62298 | German shepherd | CLEAR | M | 26/07/16 | 07/01/21 | 1626 |
| 62336 | Czechoslovakian wolfdog | CLEAR | M | 10/12/18 | 05/02/21 | 788 |
| 62351 | German shepherd | CARRIER | F | 20/12/18 | 29/01/21 | 771 |
| 62399 | Australian shepherd | CLEAR | F | 18/02/19 | 09/02/21 | 722 |
| 62417 | German shepherd | CLEAR | F | 03/11/19 | 02/02/21 | 457 |
| 62435 | Australian shepherd | CLEAR | M | 13/03/20 | 04/02/21 | 328 |
| 62436 | German shepherd | CLEAR | M | 29/12/15 | 08/02/21 | 1868 |
| 62437 | German shepherd | CLEAR | F | 29/12/15 | 08/02/21 | 1868 |
| 62438 | German shepherd | CARRIER | F | 17/11/15 | 08/02/21 | 1910 |
| 62439 | German shepherd | CLEAR | M | 24/04/15 | 08/02/21 | 2117 |
| 62466 | astore della Lessinia e del Lagor | CLEAR | | | | |
| 62470 | Rhodesian Ridgeback | CLEAR | | | | |
| 62472 | Australian shepherd | CLEAR | F | 10/05/18 | 09/02/21 | 1006 |
| 62530 | Australian shepherd | CLEAR | M | 07/02/17 | 12/02/21 | 1466 |
| 62547 | French Bulldog | CARRIER | F | 05/04/18 | 08/02/21 | 1040 |
| 62560 | German shepherd | CARRIER | F | 23/12/17 | 15/02/21 | 1150 |
| 62561 | French Bulldog | CLEAR | F | 21/09/19 | 12/02/21 | 510 |
| 62586 | French Bulldog | CLEAR | F | 07/06/19 | 13/02/21 | 617 |
| 62590 | Czechoslovakian wolfdog | CARRIER | F | 23/12/17 | 17/02/21 | 1152 |
| 62604 | German shepherd | CARRIER | M | 21/05/10 | 04/02/21 | 3912 |
| 62618 | Boston terrier | CLEAR | | | | |
| 62619 | Bernese Mountain Dog | CLEAR | F | 13/11/19 | 13/02/21 | 458 |
| 62660 | Chien de Saint-Hubert | CARRIER | F | 08/07/18 | 20/02/21 | 958 |
| 62682 | German shepherd | CARRIER | | | | |

| | | | | | | | |
|---|---|---|---|---|---|---|---|
| 62683 | Bernese Mountain Dog | CLEAR | | | | | |
| 62702 | Welsh Corgi Pembroke | CARRIER | F | 26/12/20 | 23/02/21 | 59 |
| 62703 | Welsh Corgi Pembroke | CARRIER | F | 26/12/20 | 23/02/21 | 59 |
| 62722 | Australian shepherd | CLEAR | M | 17/02/19 | 17/02/21 | 731 |
| 62723 | German shepherd | AFFECTED | M | 25/03/12 | 23/02/21 | 3257 |
| 62725 | German shepherd | CLEAR | F | 21/12/14 | 18/02/21 | 2251 |
| 62727 | Welsh Corgi Pembroke | CARRIER | F | 30/07/19 | 24/02/21 | 575 |
| 62732 | Australian shepherd | CLEAR | F | 25/04/19 | 18/02/21 | 665 |
| 62733 | Australian shepherd | CLEAR | M | 28/03/18 | 18/02/21 | 1058 |
| 62734 | Australian shepherd | CLEAR | F | 25/01/20 | 18/02/21 | 390 |
| 62754 | Bernese Mountain Dog | CLEAR | M | 10/12/18 | 25/02/21 | 808 |
| 62755 | Bernese Mountain Dog | CLEAR | F | 17/04/19 | 25/02/21 | 680 |
| 62786 | Australian shepherd | CLEAR | F | 08/09/17 | 21/02/21 | 1262 |
| 62798 | Toy poodle | CLEAR | M | 22/02/20 | 22/02/21 | 366 |
| 62803 | Czechoslovakian wolfdog | CLEAR | M | 06/02/21 | 01/03/21 | 23 |
| 62804 | Czechoslovakian wolfdog | CARRIER | M | 06/02/21 | 01/03/21 | 23 |
| 62839 | Australian shepherd | CLEAR | M | 20/02/20 | 25/02/21 | 371 |
| 62845 | Bernese Mountain Dog | CLEAR | F | 08/06/16 | 26/02/21 | 1724 |
| 62846 | Australian shepherd | CLEAR | F | 29/01/18 | 01/03/21 | 1127 |
| 62851 | Lhasa Apso | CLEAR | | | | |
| 62853 | Lhasa Apso | CLEAR | | | | |
| 62855 | Lhasa Apso | CLEAR | | | | |
| 62856 | Lhasa Apso | CLEAR | | | | |
| 62888 | Czechoslovakian wolfdog | CARRIER | M | 01/10/19 | 25/02/21 | 513 |
| 62919 | Czechoslovakian wolfdog | AFFECTED | F | 24/10/18 | 19/02/21 | 849 |
| 62923 | Barzoi | CARRIER | F | 08/11/20 | 05/03/21 | 117 |
| 62924 | Barzoi | CLEAR | M | 08/11/20 | 05/03/21 | 117 |
| 62925 | Barzoi | CARRIER | F | 08/11/20 | 05/03/21 | 117 |
| 62948 | Hovawart | CLEAR | F | 26/03/19 | 18/02/21 | 695 |
| 62981 | Toy poodle | CLEAR | F | 01/03/20 | 09/03/21 | 373 |
| 62984 | German shepherd | CLEAR | | | | |
| 63024 | Australian shepherd | CLEAR | | | | |
| 63045 | German shepherd | CLEAR | M | 25/02/20 | 12/03/21 | 381 |
| 63054 | German shepherd | CARRIER | F | 19/02/17 | 14/03/21 | 1484 |

| | | | | | | |
|---|---|---|---|---|---|---|
| 63055 | French Bulldog | CLEAR | | | | |
| 63056 | Australian shepherd | CLEAR | F | 08/07/16 | 13/03/21 | 1709 |
| 63059 | Australian shepherd | CLEAR | F | 20/11/20 | 15/03/21 | 115 |
| 63060 | Australian shepherd | CLEAR | M | 18/12/18 | 15/03/21 | 818 |
| 63061 | Australian shepherd | CARRIER | F | 20/02/19 | 15/03/21 | 754 |
| 63089 | Miniature poodle | CLEAR | F | 20/09/19 | 15/03/21 | 542 |
| 63092 | Australian shepherd | CLEAR | M | 20/03/18 | 15/03/21 | 1091 |
| 63107 | Welsh Corgi Pembroke | AFFECTED | M | 06/06/19 | 15/03/21 | 648 |
| 63118 | German shepherd | CLEAR | M | 23/02/20 | 15/03/21 | 386 |
| 63119 | Hovawart | CARRIER | F | 12/02/19 | 06/03/21 | 753 |
| 63120 | Bernese Mountain Dog | CLEAR | M | 02/11/19 | 10/02/21 | 466 |
| 63121 | Czechoslovakian wolfdog | CLEAR | M | 16/12/19 | 25/02/21 | 437 |
| 63122 | Czechoslovakian wolfdog | CLEAR | M | 16/12/19 | 11/03/21 | 451 |
| 63123 | Czechoslovakian wolfdog | CLEAR | M | 16/12/19 | 25/02/21 | 437 |
| 63138 | Shiba inu | CLEAR | | | | |
| 63232 | Welsh Corgi Pembroke | CARRIER | F | 17/09/19 | 22/03/21 | 552 |
| 63259 | Shetland shepherd | CARRIER | F | 21/05/15 | 23/03/21 | 2133 |
| 63270 | German shepherd | CLEAR | F | 31/08/17 | 24/03/21 | 1301 |
| 63271 | German shepherd | CLEAR | F | 27/02/19 | 24/03/21 | 756 |
| 63278 | Czechoslovakian wolfdog | CLEAR | F | 13/01/20 | 17/03/21 | 429 |
| 63302 | Czechoslovakian wolfdog | CLEAR | F | 30/11/19 | 22/03/21 | 478 |
| 63305 | Toy poodle | CLEAR | M | 09/12/19 | 19/03/21 | 466 |
| 63316 | French Bulldog | CARRIER | F | 26/09/15 | 16/03/21 | 1998 |
| 63322 | German shepherd | CARRIER | F | 03/06/16 | 23/03/21 | 1754 |
| 63336 | Czechoslovakian wolfdog | CLEAR | M | 29/11/16 | 22/03/21 | 1574 |
| 63337 | Welsh Corgi Pembroke | AFFECTED | F | 01/08/19 | 18/03/21 | 595 |
| 63339 | Rough Collie | AFFECTED | F | 08/02/20 | 25/03/21 | 411 |
| 63377 | Toy poodle | CLEAR | M | 30/08/17 | 30/03/21 | 1308 |
| 63406 | Belgian shepherd Malinois | CLEAR | F | 06/03/19 | 29/03/21 | 754 |
| 63415 | Bernese Mountain Dog | CARRIER | M | 29/01/16 | 30/03/21 | 1887 |
| 63456 | Welsh Corgi Pembroke | CARRIER | M | 06/01/19 | 29/03/21 | 813 |
| 63462 | Australian shepherd | CLEAR | | | | |
| 63463 | German shepherd | CLEAR | M | 20/08/20 | 29/03/21 | 221 |
| 63469 | Czechoslovakian wolfdog | AFFECTED | M | 24/10/18 | 29/03/21 | 887 |

| | | | | | | |
|---|---|---|---|---|---|---|
| 63483 | Czechoslovakian wolfdog | CLEAR | F | 07/11/19 | 01/03/21 | 480 |
| 63501 | Czechoslovakian wolfdog | CARRIER | | | | |
| 63502 | Czechoslovakian wolfdog | CARRIER | F | 06/02/21 | 06/04/21 | 59 |
| 63514 | French Bulldog | CLEAR | M | 09/02/21 | 01/04/21 | 51 |
| 63529 | Welsh Corgi Pembroke | CLEAR | M | 14/05/16 | 06/04/21 | 1788 |
| 63558 | German shepherd | CLEAR | M | 03/02/21 | 06/04/21 | 62 |
| 63635 | Australian cattledog | CLEAR | F | 20/12/15 | 09/04/21 | 1937 |
| 63637 | German shepherd | CLEAR | F | 14/01/17 | 12/04/21 | 1549 |
| 63638 | German shepherd | CLEAR | M | 13/02/14 | 12/04/21 | 2615 |
| 63647 | German shepherd | CLEAR | F | 04/04/18 | 10/04/21 | 1102 |
| 63651 | White Swiss Shepherd Dog | CLEAR | | | | |
| 63653 | Black pug dog | CARRIER | | | | |
| 63655 | Welsh Corgi Pembroke | CARRIER | M | 10/04/19 | 13/04/21 | 734 |
| 63707 | Shetland shepherd | CLEAR | M | 28/07/20 | 14/04/21 | 260 |
| 63737 | German shepherd | CLEAR | F | 23/11/15 | 15/04/21 | 1970 |
| 63738 | German shepherd | CARRIER | F | 13/07/17 | 15/04/21 | 1372 |
| 63747 | White Swiss Shepherd Dog | CLEAR | | | | |
| 63783 | Czechoslovakian wolfdog | CLEAR | | | | |
| 63888 | Toy poodle | CLEAR | F | 10/04/17 | 23/04/21 | 1474 |
| 63891 | Miniature poodle | CLEAR | M | 07/01/20 | 14/04/21 | 463 |
| 63920 | Dobermann Pinscher | CLEAR | F | 29/05/19 | 14/04/21 | 686 |
| 63921 | Dobermann Pinscher | CLEAR | M | 12/11/18 | 14/04/21 | 884 |
| 63939 | Australian shepherd | CLEAR | M | 30/07/18 | 27/04/21 | 1002 |
| 63971 | Hovawart | CARRIER | M | 14/10/19 | 24/04/21 | 558 |
| 63972 | French Bulldog | CLEAR | M | 29/12/19 | 27/04/21 | 485 |
| 63982 | Miniature poodle | CLEAR | F | 30/08/20 | 26/04/21 | 239 |
| 64003 | Bernese Mountain Dog | CLEAR | F | 14/03/19 | 26/04/21 | 774 |
| 64037 | German shepherd | CARRIER | M | 20/02/17 | 28/04/21 | 1528 |
| 64068 | German shepherd | CLEAR | M | 12/06/16 | 29/04/21 | 1782 |
| 64075 | Miniature poodle | CLEAR | F | 14/01/20 | 29/04/21 | 471 |
| 64076 | Australian shepherd | CLEAR | F | 10/04/19 | 28/04/21 | 749 |
| 64090 | Australian shepherd | CLEAR | M | 17/02/20 | 29/04/21 | 437 |
| 64104 | Belgian shepherd Groenendael | CLEAR | | | | |
| 64113 | Welsh Corgi Pembroke | AFFECTED | F | 02/10/18 | 04/05/21 | 945 |

| | | | | | | |
|---|---|---|---|---|---|---|
| 64118 | White Swiss Shepherd Dog | AFFECTED | M | 01/04/12 | 26/04/21 | 3312 |
| 64160 | Cavalier King Charles Spaniel | CARRIER | F | 15/12/15 | 04/05/21 | 1967 |
| 64163 | Welsh Corgi Pembroke | CARRIER | F | 03/04/21 | 05/05/21 | 32 |
| 64164 | Welsh Corgi Pembroke | CARRIER | M | 03/04/21 | 05/05/21 | 32 |
| 64165 | Bernese Mountain Dog | CLEAR | F | 19/09/19 | 27/04/21 | 586 |
| 64170 | Toy poodle | CLEAR | M | 22/03/20 | 04/05/21 | 408 |
| 64172 | Bernese Mountain Dog | CLEAR | | | | |
| 64219 | White Swiss Shepherd Dog | CLEAR | | | | |
| 64220 | White Swiss Shepherd Dog | CLEAR | F | 13/10/17 | 03/05/21 | 1298 |
| 64255 | German shepherd | CLEAR | F | 20/05/16 | 08/05/21 | 1814 |
| 64260 | German shepherd | CARRIER | F | 16/03/16 | 26/03/21 | 1836 |
| 64282 | German shepherd | CLEAR | F | 27/01/20 | 11/05/21 | 470 |
| 64316 | German shepherd | CLEAR | F | 21/09/16 | 12/05/21 | 1694 |
| 64339 | Australian shepherd | CLEAR | M | 24/12/19 | 07/05/21 | 500 |
| 64365 | French Bulldog | CARRIER | F | 27/07/17 | 12/05/21 | 1385 |
| 64372 | Czechoslovakian wolfdog | CARRIER | M | 11/12/17 | 17/05/21 | 1253 |
| 64400 | astore della Lessinia e del Lagor | CARRIER | | | | |
| 64423 | Czechoslovakian wolfdog | CLEAR | M | 18/02/16 | 17/05/21 | 1915 |
| 64424 | German shepherd | CARRIER | | | | |
| 64441 | German shepherd | CARRIER | | | | |
| 64454 | Australian shepherd | CLEAR | M | 10/04/19 | 20/05/21 | 771 |
| 64499 | Australian shepherd | CLEAR | F | 16/06/17 | 17/05/21 | 1431 |
| 64556 | Welsh Corgi Pembroke | AFFECTED | F | 30/10/20 | 24/05/21 | 206 |
| 64558 | Welsh Corgi Pembroke | CLEAR | F | 01/03/21 | 24/05/21 | 84 |
| 64617 | Welsh Corgi Pembroke | CLEAR | F | 08/04/21 | 27/05/21 | 49 |
| 64618 | Welsh Corgi Pembroke | CLEAR | M | 08/04/21 | 27/05/21 | 49 |
| 64619 | Welsh Corgi Pembroke | AFFECTED | F | 08/04/21 | 27/05/21 | 49 |
| 64620 | Welsh Corgi Pembroke | CARRIER | M | 08/04/21 | 27/05/21 | 49 |
| 64621 | Welsh Corgi Pembroke | CLEAR | F | 08/04/21 | 27/05/21 | 49 |
| 64646 | Australian shepherd | CLEAR | F | 01/01/20 | 24/05/21 | 509 |
| 64647 | Australian shepherd | CARRIER | F | 02/05/20 | 24/05/21 | 387 |
| 64653 | Australian shepherd | CLEAR | F | 20/11/20 | 01/06/21 | 193 |
| 64674 | German shepherd | CLEAR | F | 04/05/16 | 27/05/21 | 1849 |
| 64680 | Belgian shepherd Malinois | CLEAR | | | | |

| | | | | | | |
|---|---|---|---|---|---|---|
| 64707 | Australian shepherd | CLEAR | F | 16/03/19 | 20/05/21 | 796 |
| 64738 | German shepherd | CARRIER | M | 29/10/20 | 29/05/21 | 212 |
| 64739 | Bernese Mountain Dog | CLEAR | F | 18/03/18 | 03/06/21 | 1173 |
| 64792 | French Bulldog | CLEAR | M | 04/09/20 | 31/05/21 | 269 |
| 64800 | Czechoslovakian wolfdog | CLEAR | M | 17/01/20 | 07/06/21 | 507 |
| 64893 | Rough Collie | AFFECTED | M | 01/02/18 | 12/06/21 | 1227 |
| 64894 | Rough Collie | CLEAR | F | 01/02/18 | 12/06/21 | 1227 |
| 64895 | Czechoslovakian wolfdog | CLEAR | | | | |
| 64896 | Czechoslovakian wolfdog | CLEAR | | | | |
| 64897 | German shepherd | CLEAR | | | | |
| 64917 | Rough Collie | CARRIER | F | 08/02/20 | 12/06/21 | 490 |
| 64946 | French Bulldog | CARRIER | F | 23/05/20 | 13/06/21 | 386 |
| 64954 | Barzoi | CARRIER | | | | |
| 64957 | Boston terrier | CLEAR | | | | |
| 64997 | French Bulldog | CLEAR | | | | |
| 65057 | German shepherd | CLEAR | | | | |
| 65059 | Welsh Corgi Pembroke | CLEAR | F | 08/04/21 | 10/06/21 | 63 |
| 65060 | Welsh Corgi Pembroke | CLEAR | M | 08/04/21 | 10/06/21 | 63 |
| 65065 | French Bulldog | CLEAR | F | 23/06/19 | 15/06/21 | 723 |
| 65086 | Hovawart | CLEAR | M | 05/04/19 | 14/06/21 | 801 |
| 65087 | Cavalier King Charles Spaniel | AFFECTED | F | 21/04/19 | 14/06/21 | 785 |
| 65088 | Cavalier King Charles Spaniel | CARRIER | F | 02/02/20 | 14/06/21 | 498 |
| 65199 | Hovawart | CARRIER | F | 23/12/08 | 17/06/21 | 4559 |
| 65201 | German shepherd | CLEAR | F | 20/02/17 | 15/06/21 | 1576 |
| 65202 | German shepherd | CLEAR | | | | |
| 65216 | Welsh Corgi Pembroke | AFFECTED | F | 16/08/20 | 18/06/21 | 306 |
| 65217 | Welsh Corgi Pembroke | AFFECTED | | | | |
| 65223 | German shepherd | CARRIER | F | 15/03/20 | 21/06/21 | 463 |
| 65224 | German shepherd | CARRIER | M | 25/05/16 | 21/06/21 | 1853 |
| 65225 | German shepherd | CARRIER | F | 15/06/16 | 21/06/21 | 1832 |
| 65310 | Australian shepherd | CLEAR | M | 24/02/21 | 22/06/21 | 118 |
| 65323 | Shetland shepherd | CLEAR | F | 03/03/21 | 22/06/21 | 111 |
| 65329 | Rottweiler | CLEAR | F | 25/12/17 | 23/06/21 | 1276 |
| 65407 | German shepherd | AFFECTED | M | 14/10/18 | 28/06/21 | 988 |

| | | | | | | | |
|---|---|---|---|---|---|---|---|
| 65416 | Czechoslovakian wolfdog | AFFECTED | M | 16/01/10 | 29/06/21 | 4182 |
| 65430 | White Swiss Shepherd Dog | CLEAR | | | | |
| 65442 | Australian shepherd | CLEAR | F | 12/05/19 | 02/06/21 | 752 |
| 65459 | Toy poodle | CLEAR | F | 09/04/18 | 29/06/21 | 1177 |
| 65460 | Toy poodle | CLEAR | M | 23/12/20 | 29/06/21 | 188 |
| 65461 | Toy poodle | CLEAR | M | 27/07/20 | 29/06/21 | 337 |
| 65462 | Toy poodle | CLEAR | F | 03/12/20 | 29/06/21 | 208 |
| 65463 | Toy poodle | CLEAR | F | 03/12/20 | 29/06/21 | 208 |
| 65464 | Toy poodle | CLEAR | F | 23/12/20 | 29/06/21 | 188 |
| 65465 | Toy poodle | CLEAR | F | 05/11/20 | 29/06/21 | 236 |
| 65466 | Toy poodle | CLEAR | F | 23/12/20 | 29/06/21 | 188 |
| 65467 | Toy poodle | CLEAR | F | 18/01/19 | 29/06/21 | 893 |
| 65468 | Toy poodle | CLEAR | F | 14/04/18 | 29/06/21 | 1172 |
| 65469 | Toy poodle | CLEAR | F | 29/04/19 | 29/06/21 | 792 |
| 65470 | Toy poodle | CARRIER | F | 19/01/17 | 29/06/21 | 1622 |
| 65471 | Toy poodle | CARRIER | F | 14/05/20 | 29/06/21 | 411 |
| 65472 | Toy poodle | CARRIER | M | 14/04/18 | 29/06/21 | 1172 |
| 65473 | Toy poodle | CARRIER | F | 20/05/19 | 29/06/21 | 771 |
| 65474 | Toy poodle | CARRIER | F | 14/05/20 | 29/06/21 | 411 |
| 65475 | Toy poodle | CLEAR | F | 19/07/20 | 29/06/21 | 345 |
| 65513 | Poodle | CLEAR | F | 13/01/20 | 03/07/21 | 537 |
| 65534 | Cavalier King Charles Spaniel | CLEAR | F | 06/06/20 | 03/07/21 | 392 |
| 65549 | German shepherd | CLEAR | M | 27/01/13 | 01/07/21 | 3077 |
| 65563 | Czechoslovakian wolfdog | CLEAR | | | | |
| 65568 | Rough Collie | CARRIER | F | 07/04/18 | 04/07/21 | 1184 |
| 65570 | Cavalier King Charles Spaniel | CARRIER | F | 11/09/19 | 04/07/21 | 662 |
| 65573 | White Swiss Shepherd Dog | CLEAR | | | | |
| 65575 | Rough Collie | CLEAR | | | | |
| 65588 | Poodle | CLEAR | F | 25/02/18 | 04/07/21 | 1225 |
| 65589 | Dobermann Pinscher | CLEAR | F | 09/07/19 | 04/07/21 | 726 |
| 65646 | Czechoslovakian wolfdog | CARRIER | F | 26/01/18 | 24/06/21 | 1245 |
| 65662 | Czechoslovakian wolfdog | CARRIER | F | 18/10/16 | 18/06/21 | 1704 |
| 65666 | Bernese Mountain Dog | CLEAR | F | 21/11/17 | 01/07/21 | 1318 |
| 65668 | Bernese Mountain Dog | CLEAR | M | 09/05/17 | 05/07/21 | 1518 |

| | | | | | | |
|---|---|---|---|---|---|---|
| 65697 | French Bulldog | CLEAR | F | 01/02/20 | 03/07/21 | 518 |
| 65721 | Belgian shepherd Groenendael | CLEAR | M | 13/06/18 | 07/07/21 | 1120 |
| 65797 | Mixed breed | CARRIER | M | 01/05/07 | 10/07/21 | 5184 |
| 65808 | German shepherd | CARRIER | F | 05/07/10 | 08/07/21 | 4021 |
| 65829 | German shepherd | CLEAR | M | 12/09/12 | 12/07/21 | 3225 |
| 65911 | Toy poodle | CLEAR | F | 18/08/18 | 13/07/21 | 1060 |
| 65912 | Toy poodle | CARRIER | F | 28/09/19 | 13/07/21 | 654 |
| 65913 | Toy poodle | CARRIER | M | 20/06/18 | 13/07/21 | 1119 |
| 65915 | Toy poodle | CLEAR | M | 19/01/20 | 14/07/21 | 542 |
| 65916 | Poodle | CLEAR | | | | |
| 66003 | French Bulldog | CLEAR | M | 07/12/18 | 25/06/21 | 931 |
| 66084 | Rough Collie | AFFECTED | | | | |
| 66088 | Czechoslovakian wolfdog | CARRIER | F | 13/05/20 | 21/07/21 | 434 |
| 66089 | Czechoslovakian wolfdog | CLEAR | M | 23/06/19 | 21/07/21 | 759 |
| 66104 | Bernese Mountain Dog | CLEAR | | | | |
| 66146 | Australian shepherd | CLEAR | F | 16/04/21 | 27/07/21 | 102 |
| 66174 | French Bulldog | CARRIER | F | 16/05/20 | 26/07/21 | 436 |
| 66227 | Parson Russell Terrier | CLEAR | | | | |
| 66236 | Shetland shepherd | CLEAR | F | 19/12/20 | 31/07/21 | 224 |
| 66237 | Shetland shepherd | CLEAR | F | 19/12/20 | 31/07/21 | 224 |
| 66264 | Australian shepherd | CLEAR | M | 15/11/19 | 31/07/21 | 624 |
| 66295 | Australian shepherd | CLEAR | F | 05/04/21 | 02/08/21 | 119 |
| 66317 | Barzoi | CLEAR | F | 05/10/10 | 01/08/21 | 3953 |
| 66318 | Australian shepherd | CLEAR | F | 18/04/19 | 03/08/21 | 838 |
| 66392 | German shepherd | CLEAR | | | | |
| 66476 | Australian shepherd | CLEAR | M | 26/04/20 | 10/08/21 | 471 |
| 66492 | German shepherd | CLEAR | M | 01/03/12 | 06/08/21 | 3445 |
| 66508 | Czechoslovakian wolfdog | CLEAR | | | | |
| 66544 | Bernese Mountain Dog | CLEAR | | | | |
| 66672 | astore della Lessinia e del Lagor | CLEAR | | | | |
| 66677 | Czechoslovakian wolfdog | CARRIER | M | 16/03/15 | 20/08/21 | 2349 |
| 66758 | Dobermann Pinscher | CLEAR | F | 10/07/20 | 30/08/21 | 416 |
| 66778 | French Bulldog | CLEAR | F | 25/01/20 | 27/08/21 | 580 |
| 66809 | Mixed breed | CARRIER | F | 01/09/09 | 31/08/21 | 4382 |

| | | | | | | |
|---|---|---|---|---|---|---|
| 66810 | German shepherd | CARRIER | M | 04/04/13 | 31/08/21 | 3071 |
| 66852 | Czechoslovakian wolfdog | CLEAR | F | 06/05/20 | 06/09/21 | 488 |
| 66854 | Australian shepherd | CLEAR | F | 27/02/19 | 01/09/21 | 917 |
| 66855 | Australian shepherd | CLEAR | M | 11/02/17 | 01/09/21 | 1663 |
| 66856 | Australian shepherd | CARRIER | F | 26/08/17 | 01/09/21 | 1467 |
| 66881 | German shepherd | AFFECTED | | | | |
| 66959 | French Bulldog | CLEAR | F | 22/06/18 | 07/09/21 | 1173 |
| 66985 | astore della Lessinia e del Lagor | CLEAR | | | | |
| 67002 | German shepherd | CARRIER | | | | |
| 67048 | German shepherd | CLEAR | | | | |
| 67057 | Toy poodle | AFFECTED | | | | |
| 67058 | Toy poodle | CLEAR | | | | |
| 67059 | Toy poodle | CARRIER | | | | |
| 67079 | Canaan dog | CARRIER | M | 19/12/14 | 15/09/21 | 2462 |
| 67086 | Australian cattledog | CLEAR | F | 11/03/19 | 15/09/21 | 919 |
| 67110 | Poodle | CLEAR | F | 15/12/19 | 18/09/21 | 643 |
| 67114 | Poodle | CLEAR | F | 01/03/21 | 18/09/21 | 201 |
| 67115 | Poodle | CLEAR | M | 30/04/18 | 18/09/21 | 1237 |
| 67127 | Australian shepherd | CLEAR | M | 19/08/19 | 18/09/21 | 761 |
| 67144 | Barzoi | CARRIER | M | 22/04/19 | 18/09/21 | 880 |
| 67207 | Welsh Corgi Pembroke | AFFECTED | M | 25/03/19 | 16/09/21 | 906 |
| 67273 | Dogo argentino | CLEAR | F | 15/08/14 | 20/09/21 | 2593 |
| 67325 | Bernese Mountain Dog | CARRIER | F | 14/07/17 | 22/09/21 | 1531 |
| 67373 | Belgian shepherd Malinois | CLEAR | | | | |
| 67399 | Australian shepherd | CLEAR | F | 14/08/18 | 24/09/21 | 1137 |
| 67443 | Black pug dog | CARRIER | M | 11/04/18 | 28/09/21 | 1266 |
| 67451 | Czechoslovakian wolfdog | CLEAR | M | 09/08/20 | 23/09/21 | 410 |
| 67480 | French Bulldog | AFFECTED | M | 14/09/20 | 28/09/21 | 379 |
| 67482 | Australian shepherd | CLEAR | F | 21/03/20 | 28/09/21 | 556 |
| 67486 | German shepherd | CARRIER | M | 10/09/17 | 24/09/21 | 1475 |
| 67521 | Mixed breed | AFFECTED | F | 19/10/11 | 27/09/21 | 3631 |
| 67540 | Bernese Mountain Dog | CLEAR | | | | |
| 67551 | German shepherd | CARRIER | F | 07/09/14 | 04/10/21 | 2584 |
| 67606 | German shepherd | CARRIER | | | | |

| | | | | | | |
|---|---|---|---|---|---|---|
| 67608 | German shepherd | CLEAR | F | 29/06/17 | 04/10/21 | 1558 |
| 67641 | German shepherd | CLEAR | M | 06/02/20 | 06/10/21 | 608 |
| 67749 | Miniature poodle | CLEAR | M | 01/02/20 | 11/10/21 | 618 |
| 67756 | German shepherd | CLEAR | F | 10/04/20 | 13/10/21 | 551 |
| 67776 | Czechoslovakian wolfdog | CLEAR | M | 20/01/19 | 06/10/21 | 990 |
| 67820 | White Swiss Shepherd Dog | CLEAR | | | | |
| 67837 | Rough Collie | CARRIER | | | | |
| 67890 | Dutch Shepherd Dog | CLEAR | | | | |
| 67897 | Rottweiler | CLEAR | M | 16/12/19 | 08/10/21 | 662 |
| 67898 | Rottweiler | CLEAR | F | 28/10/20 | 08/10/21 | 345 |
| 67910 | Belgian shepherd Groenendael | CLEAR | F | 08/10/20 | 15/10/21 | 372 |
| 67925 | Alaskan Malamute | CLEAR | | | | |
| 67952 | Bernese Mountain Dog | CLEAR | | | | |
| 67984 | Dogue de Bordeaux | CLEAR | | | | |
| 67985 | Dogue de Bordeaux | CLEAR | | | | |
| 68034 | German shepherd | CARRIER | F | 02/07/19 | 11/10/21 | 832 |
| 68036 | French Bulldog | CLEAR | F | 16/06/19 | 19/10/21 | 856 |
| 68082 | Apuan Alps' sheperd | AFFECTED | | | | |
| 68086 | Apuan Alps' sheperd | AFFECTED | | | | |
| 68089 | Apuan Alps' sheperd | CARRIER | | | | |
| 68138 | Welsh Corgi Pembroke | AFFECTED | | | | |
| 68147 | Australian cattledog | CLEAR | F | 15/06/21 | 18/10/21 | 125 |
| 68198 | Cao de Agua Portugues | CLEAR | | | | |
| 68204 | German shepherd | AFFECTED | F | 06/10/12 | 27/10/21 | 3308 |
| 68228 | Greyhound | CLEAR | | | | |
| 68238 | Bobtail | CLEAR | F | 04/04/19 | 30/10/21 | 940 |
| 68243 | White Swiss Shepherd Dog | CLEAR | | | | |
| 68270 | Welsh Corgi Cardigan | CLEAR | | | | |
| 68283 | Shiba inu | CLEAR | | | | |
| 68322 | Dobermann Pinscher | CLEAR | | 30/03/21 | 01/11/21 | 216 |
| 68378 | Poodle | CLEAR | M | 09/10/20 | 03/11/21 | 390 |
| 68392 | Dobermann Pinscher | CLEAR | M | 22/07/20 | 03/11/21 | 469 |
| 68408 | Bernese Mountain Dog | CLEAR | | | | |
| 68467 | Welsh Corgi Pembroke | CARRIER | M | 28/04/20 | 04/11/21 | 555 |

| | | | | | | |
|---|---|---|---|---|---|---|
| 68474 | French Bulldog | CLEAR | M | 23/05/21 | 03/11/21 | 164 |
| 68565 | White Swiss Shepherd Dog | CLEAR | | | | |
| 68662 | Barzoi | CLEAR | M | 23/11/19 | 09/11/21 | 717 |
| 68663 | Barzoi | CLEAR | M | 23/11/19 | 09/11/21 | 717 |
| 68683 | Rhodesian Ridgeback | | M | 21/12/20 | 13/11/21 | 327 |
| 68688 | German shepherd | CLEAR | M | 08/03/18 | 13/11/21 | 1346 |
| 68689 | Poodle | CLEAR | M | 25/08/19 | 13/11/21 | 811 |
| 68696 | Poodle | CLEAR | | | | |
| 68698 | Poodle | CLEAR | | | | |
| 68699 | Poodle | CLEAR | | | | |
| 68700 | Poodle | CLEAR | | | | |
| 68701 | Poodle | CLEAR | | | | |
| 68721 | Bernese Mountain Dog | CLEAR | | | | |
| 68732 | Poodle | CARRIER | | | | |
| 68747 | Toy poodle | CLEAR | | | | |
| 68757 | Hovawart | CLEAR | F | 20/10/20 | 26/10/21 | 371 |
| 68794 | Czechoslovakian wolfdog | CLEAR | F | 27/10/20 | 05/11/21 | 374 |
| 68806 | French Bulldog | CARRIER | | | | |
| 68833 | Poodle | CLEAR | | | | |
| 68850 | French Bulldog | CARRIER | F | 27/02/20 | 15/11/21 | 627 |
| 68932 | German shepherd | CARRIER | F | 27/09/20 | 16/11/21 | 415 |
| 68935 | Poodle | CLEAR | F | 25/07/19 | 23/11/21 | 852 |
| 68938 | Rough Collie | CARRIER | F | 02/10/21 | 18/11/21 | 47 |
| 68941 | German shepherd | CLEAR | F | 10/11/20 | 16/11/21 | 371 |
| 68945 | Shiba inu | CLEAR | M | 10/11/20 | 22/11/21 | 377 |
| 68955 | Miniature poodle | CLEAR | M | | 22/11/21 | |
| 68998 | Bolognese | CLEAR | | | | |
| 69022 | American akita | CLEAR | F | 05/03/16 | 15/11/21 | 2081 |
| 69039 | Australian shepherd | CARRIER | F | 25/05/21 | 22/11/21 | 181 |
| 69076 | French Bulldog | CARRIER | M | 17/11/20 | 25/11/21 | 373 |
| 69079 | German shepherd | CLEAR | M | 21/11/20 | 25/11/21 | 369 |
| 69084 | German shepherd | CARRIER | F | 26/02/21 | 27/11/21 | 274 |
| 69094 | Dobermann Pinscher | CLEAR | M | 12/09/19 | 11/11/21 | 791 |
| 69096 | Australian shepherd | CLEAR | M | 26/10/20 | 29/11/21 | 399 |

| | | | | | | |
|---|---|---|---|---|---|---|
| 69099 | Bernese Mountain Dog | CLEAR | M | 04/04/19 | 29/11/21 | 970 |
| 69125 | French Bulldog | CLEAR | M | 18/11/20 | 04/12/21 | 381 |
| 69127 | Czechoslovakian wolfdog | CLEAR | M | 19/10/20 | 04/12/21 | 411 |
| 69136 | Welsh Corgi Pembroke | AFFECTED | M | 29/06/20 | 04/12/21 | 523 |
| 69211 | White Swiss Shepherd Dog | CLEAR | F | 15/12/20 | 05/12/21 | 355 |
| 69247 | Basset hound | CLEAR | F | 30/01/21 | 05/12/21 | 309 |
| 69270 | Australian shepherd | CLEAR | M | 05/08/20 | 25/11/21 | 477 |
| 69271 | Australian shepherd | CLEAR | F | 06/09/20 | 25/11/21 | 445 |
| 69281 | White Swiss Shepherd Dog | CLEAR | F | 12/02/21 | 30/11/21 | 291 |
| 69303 | German shepherd | CARRIER | M | 21/08/20 | 30/11/21 | 466 |
| 69311 | Welsh Corgi Pembroke | AFFECTED | F | 09/11/19 | 06/12/21 | 758 |
| 69318 | Welsh Corgi Pembroke | CARRIER | M | 01/02/15 | 01/12/21 | 2495 |
| 69332 | German shepherd | CLEAR | M | 11/06/17 | 06/12/21 | 1639 |
| 69388 | Australian shepherd | CARRIER | M | 01/06/16 | 24/11/21 | 2002 |
| 69398 | Shetland shepherd | CLEAR | M | 18/04/21 | 02/12/21 | 228 |
| 69416 | Poodle | CLEAR | M | 01/10/20 | 06/12/21 | 431 |
| 69424 | Poodle | CLEAR | M | 14/03/21 | 07/12/21 | 268 |
| 69484 | Czechoslovakian wolfdog | CLEAR | F | 27/08/20 | 04/11/21 | 434 |
| 69574 | Czechoslovakian wolfdog | CLEAR | | | | |
| 69621 | German shepherd | AFFECTED | F | 11/01/21 | 16/02/21 | 36 |
| 69658 | Toy poodle | CLEAR | M | 23/08/17 | 29/12/21 | 1589 |
| 69666 | Australian shepherd | CARRIER | F | 16/11/18 | 27/12/21 | 1137 |
| 69677 | Czechoslovakian wolfdog | CARRIER | M | 19/10/20 | 21/12/21 | 428 |
| 69742 | Poodle | CLEAR | M | 19/02/21 | 31/12/21 | 315 |
| 69754 | Mixed breed | CLEAR | F | | 29/12/21 | |
| 69755 | Mixed breed | CLEAR | F | | 29/12/21 | |
| 69767 | German shepherd | CARRIER | M | 21/08/20 | 30/11/21 | 466 |
| 69793 | German shepherd | CLEAR | F | 19/09/20 | 04/01/22 | 472 |
| 69816 | Hovawart | CLEAR | M | 04/10/21 | 01/10/22 | 362 |
| 69819 | Medium poodle | CARRIER | m | 11/09/16 | 10/01/22 | 1947 |
| 69826 | Australian shepherd | CLEAR | M | 25/07/18 | 10/01/22 | 1265 |
| 69831 | Czechoslovakian wolfdog | CLEAR | F | 18/03/19 | 10/01/22 | 1029 |
| 69832 | French Bulldog | AFFECTED | F | 20/07/20 | 08/01/22 | 537 |
| 69931 | French Bulldog | CLEAR | M | 27/01/21 | 11/01/22 | 349 |

| | | | | | | |
|---|---|---|---|---|---|---|
| 69935 | German shepherd | CLEAR | F | 11/05/19 | 31/12/21 | 965 |
| 69936 | German shepherd | CLEAR | F | 18/11/21 | 03/01/22 | 46 |
| 69937 | German shepherd | CARRIER | F | 18/11/21 | 03/01/22 | 46 |
| 69938 | German shepherd | CLEAR | F | 18/11/21 | 03/01/22 | 46 |
| 69939 | German shepherd | CARRIER | F | 18/11/21 | 03/01/22 | 46 |
| 69948 | Poodle | CLEAR | F | 10/03/20 | 14/01/22 | 675 |
| 69955 | Czechoslovakian wolfdog | CLEAR | F | 01/03/21 | 13/01/22 | 318 |
| 69964 | Czechoslovakian wolfdog | AFFECTED | M | 26/11/12 | 14/01/22 | 3336 |
| 69991 | Poodle | CLEAR | F | 23/12/19 | 15/01/22 | 754 |
| 69992 | Toy poodle | CLEAR | M | 18/11/19 | 15/01/22 | 789 |
| 69993 | Miniature poodle | CLEAR | F | 26/01/18 | 15/01/22 | 1450 |
| 69994 | Miniature poodle | CLEAR | M | 02/11/17 | 15/01/22 | 1535 |
| 69995 | Poodle | CLEAR | F | 23/12/19 | 15/01/22 | 754 |
| 70019 | White Swiss Shepherd Dog | CLEAR | F | 17/11/18 | 13/01/22 | 1153 |
| 70020 | White Swiss Shepherd Dog | CLEAR | M | 05/09/15 | 13/01/22 | 2322 |
| 70033 | Cavalier King Charles Spaniel | CLEAR | F | 28/11/19 | 18/01/22 | 782 |
| 70034 | Australian cattledog | CLEAR | F | 30/04/19 | 17/01/22 | 993 |
| 70035 | Australian cattledog | CLEAR | M | 30/11/20 | 17/01/22 | 413 |
| 70040 | Rough Collie | AFFECTED | F | 28/04/20 | 15/01/22 | 627 |
| 70042 | Toy poodle | CLEAR | M | 10/01/16 | 17/01/22 | 2199 |
| 70065 | Scotch Collie | AFFECTED | M | 05/06/12 | 13/01/21 | 3144 |
| 70100 | Australian shepherd | CLEAR | F | 21/12/19 | 21/01/22 | 762 |
| 70120 | Australian shepherd | CLEAR | M | 06/05/18 | 19/01/22 | 1354 |
| 70121 | Australian shepherd | CLEAR | F | 06/04/21 | 19/01/22 | 288 |
| 70161 | Hovawart | CLEAR | F | 13/06/15 | 24/01/22 | 2417 |
| 70171 | Shetland shepherd | CLEAR | M | 28/03/19 | 24/01/22 | 1033 |
| 70180 | German shepherd | CLEAR | F | 18/12/20 | 25/01/22 | 403 |
| 70181 | German shepherd | CARRIER | M | 20/09/18 | 25/01/22 | 1223 |
| 70204 | Czechoslovakian wolfdog | CLEAR | M | 02/11/20 | 25/01/22 | 449 |
| 70234 | Shih tzu | CLEAR | F | 04/06/09 | 24/01/22 | 4617 |
| 70252 | Australian shepherd | CLEAR | F | 03/10/20 | 27/01/22 | 481 |
| 70253 | Australian shepherd | CLEAR | M | 18/02/20 | 27/01/22 | 709 |
| 70254 | Australian shepherd | CLEAR | F | 09/08/20 | 27/01/22 | 536 |
| 70255 | Australian shepherd | CARRIER | M | 21/07/20 | 27/01/22 | 555 |

| | | | | | | |
|---|---|---|---|---|---|---|
| 70256 | Australian shepherd | CLEAR | F | 20/11/19 | 27/01/22 | 799 |
| 70257 | Australian shepherd | CLEAR | F | 20/02/20 | 27/01/22 | 707 |
| 70259 | Cavalier King Charles Spaniel | CARRIER | M | 04/07/20 | 26/01/22 | 571 |
| 70300 | German shepherd | CLEAR | F | 02/08/14 | 26/01/22 | 2734 |
| 70308 | Czechoslovakian wolfdog | CLEAR | F | 31/12/17 | 25/01/22 | 1486 |
| 70488 | Bernese Mountain Dog | CLEAR | F | 31/03/15 | 02/02/22 | 2500 |
| 70528 | Australian shepherd | CLEAR | F | 17/07/20 | 01/02/22 | 564 |
| 70561 | Maltese | CLEAR | F | 01/01/20 | 04/02/22 | 765 |
| 70562 | Maltese | CLEAR | M | 01/01/20 | 02/02/22 | 763 |
| 70564 | Maltese | CLEAR | F | 20/02/21 | 02/04/22 | 406 |
| 70567 | Maltese | CLEAR | M | 11/03/18 | 04/02/22 | 1426 |
| 70568 | Maltese | CLEAR | F | 01/01/20 | 04/02/22 | 765 |
| 70569 | Maltese | CLEAR | F | 09/01/20 | 04/02/22 | 757 |
| 70570 | Maltese | CLEAR | F | 20/02/21 | 04/02/22 | 349 |
| 70571 | Maltese | CLEAR | F | 09/01/20 | 04/02/22 | 757 |
| 70572 | Maltese | CLEAR | F | 09/01/20 | 04/02/22 | 757 |
| 70606 | German shepherd | CARRIER | F | 14/08/15 | 04/02/22 | 2366 |
| 70607 | Czechoslovakian wolfdog | CARRIER | F | 12/12/21 | 31/01/22 | 50 |
| 70630 | Poodle | CARRIER | | | | |
| 70651 | Czechoslovakian wolfdog | CLEAR | M | 19/02/20 | 07/02/22 | 719 |
| 70660 | German shepherd | CLEAR | M | 11/05/12 | 04/02/22 | 3556 |
| 70664 | Belgian shepherd Malinois | CLEAR | M | 03/08/20 | 02/02/22 | 548 |
| 70694 | French Bulldog | CLEAR | F | 01/01/20 | 10/02/22 | 771 |
| 70748 | Rhodesian Ridgeback | CLEAR | F | 25/04/19 | 14/02/22 | 1026 |
| 70812 | Shetland shepherd | CARRIER | M | 19/03/13 | 16/02/22 | 3256 |
| 70889 | Czechoslovakian wolfdog | CLEAR | F | 17/11/17 | 18/02/22 | 1554 |
| 70897 | Greyhound | CLEAR | M | 01/07/19 | 17/02/22 | 962 |
| 70913 | Barzoi | CLEAR | F | 27/10/21 | 21/02/22 | 117 |
| 70925 | Poodle | CLEAR | M | 23/03/21 | 18/02/22 | 332 |
| 70995 | Barzoi | CARRIER | F | 27/10/21 | 22/02/22 | 118 |
| 71000 | Shetland shepherd | CLEAR | M | 03/03/21 | 15/02/22 | 349 |
| 71004 | Czechoslovakian wolfdog | CLEAR | M | 14/02/21 | 22/02/22 | 373 |
| 71032 | French Bulldog | CLEAR | F | 20/03/20 | 21/02/22 | 703 |
| 71039 | White Swiss Shepherd Dog | CLEAR | F | 25/03/19 | 23/02/22 | 1066 |

| | | | | | | |
|---|---|---|---|---|---|---|
| 71048 | Australian shepherd | CLEAR | F | 30/09/21 | 24/02/22 | 147 |
| 71075 | Bobtail | CLEAR | F | 15/03/20 | 25/02/22 | 712 |
| 71088 | Czechoslovakian wolfdog | CLEAR | F | 02/11/20 | 12/02/22 | 467 |
| 71114 | Australian shepherd | CLEAR | F | 27/11/19 | 28/02/22 | 824 |
| 71117 | Australian shepherd | CLEAR | M | 05/04/21 | 27/02/22 | 328 |
| 71118 | Australian shepherd | CLEAR | M | 05/04/21 | 27/02/22 | 328 |
| 71128 | French Bulldog | CARRIER | F | 21/10/20 | 28/02/22 | 495 |
| 71176 | Shetland shepherd | CLEAR | M | 23/12/21 | 04/03/22 | 71 |
| 71227 | Bernese Mountain Dog | AFFECTED | F | 14/01/14 | 02/03/22 | 2969 |
| 71238 | German shepherd | CARRIER | M | 10/02/19 | 03/03/22 | 1117 |
| 71245 | Welsh Corgi Pembroke | CARRIER | F | 01/01/20 | 02/03/22 | 791 |
| 71284 | Tibetan spaniel | CLEAR | M | 14/10/18 | 08/03/22 | 1241 |
| 71285 | Bernese Mountain Dog | CLEAR | | | 08/03/22 | |
| 71287 | Cavalier King Charles Spaniel | CLEAR | F | 20/02/19 | 07/03/22 | 1111 |
| 71320 | Dutch Shepherd Dog | CLEAR | F | 30/07/20 | 09/03/22 | 587 |
| 71358 | Czechoslovakian wolfdog | CLEAR | M | 25/03/19 | 09/03/22 | 1080 |
| 71359 | Welsh Corgi Pembroke | CARRIER | M | 22/03/21 | 09/03/22 | 352 |
| 71383 | Barzoi | CLEAR | M | 05/09/17 | 13/03/22 | 1650 |
| 71401 | Bernese Mountain Dog | CARRIER | M | 27/11/20 | 12/03/22 | 470 |
| 71414 | German shepherd | CLEAR | F | 24/04/20 | 11/03/22 | 686 |
| 71426 | Welsh Corgi Pembroke | CLEAR | M | 20/01/22 | 14/03/22 | 53 |
| 71427 | Welsh Corgi Pembroke | CLEAR | F | 20/01/22 | 14/03/22 | 53 |
| 71428 | Welsh Corgi Pembroke | CLEAR | F | 20/01/22 | 14/03/22 | 53 |
| 71429 | Welsh Corgi Pembroke | CLEAR | F | 20/01/22 | 14/03/22 | 53 |
| 71448 | Australian shepherd | CARRIER | M | 28/11/15 | 14/03/22 | 2298 |
| 71464 | Hovawart | AFFECTED | M | 04/12/11 | 16/03/22 | 3755 |
| 71492 | Welsh Corgi Pembroke | AFFECTED | M | 15/07/21 | 21/02/22 | 221 |
| 71496 | Belgian shepherd Malinois | CLEAR | F | 03/05/20 | 09/03/22 | 675 |
| 71502 | German shepherd | CARRIER | F | 18/12/19 | 15/03/22 | 818 |
| 71531 | German shepherd | CLEAR | M | 20/12/18 | 14/03/22 | 1180 |
| 71603 | Belgian shepherd Groenendael | CLEAR | F | 24/02/19 | 21/03/22 | 1121 |
| 71604 | Belgian shepherd Tervueren | CLEAR | F | 30/09/17 | 21/03/22 | 1633 |
| 71611 | Czechoslovakian wolfdog | CARRIER | M | 22/09/19 | 22/03/22 | 912 |
| 71621 | Belgian shepherd Malinois | CLEAR | F | 22/04/20 | 18/03/22 | 695 |

| | | | | | | |
|---|---|---|---|---|---|---|
| 71743 | Poodle | CLEAR | F | 10/03/21 | 26/03/22 | 381 |
| 71748 | Poodle | CLEAR | M | 06/05/21 | 26/03/22 | 324 |
| 71759 | Poodle | CLEAR | F | 09/02/21 | 27/03/22 | 411 |
| 71763 | Poodle | CLEAR | M | 04/05/19 | 27/03/22 | 1058 |
| 71773 | Greyhound | CLEAR | M | 13/01/20 | 27/03/22 | 804 |
| 71788 | Hovawart | CLEAR | F | 24/02/21 | 27/03/22 | 396 |
| 71909 | German shepherd | CARRIER | M | 20/09/18 | 31/03/22 | 1288 |
| 72014 | Welsh Corgi Pembroke | AFFECTED | M | 10/03/21 | 04/04/22 | 390 |
| 72015 | Welsh Corgi Pembroke | CARRIER | F | 10/02/21 | 04/04/22 | 418 |
| 72019 | Siberian Husky | CLEAR | F | 19/06/19 | 01/04/22 | 1017 |
| 72048 | Australian shepherd | CLEAR | F | 23/01/22 | 04/06/22 | 132 |
| 72069 | French Bulldog | CLEAR | M | 30/03/21 | 06/04/22 | 372 |
| 72096 | Barzoi | CLEAR | F | 05/09/17 | 09/04/22 | 1677 |
| 72120 | Poodle | CLEAR | F | 21/03/21 | 09/04/22 | 384 |
| 72127 | Shetland shepherd | CLEAR | F | 18/06/21 | 09/04/22 | 295 |
| 72130 | Hovawart | CARRIER | M | 22/03/17 | 09/04/22 | 1844 |
| 72135 | Poodle | CLEAR | F | 19/02/21 | 09/04/22 | 414 |
| 72141 | Rottweiler | CLEAR | F | 06/10/21 | 09/04/22 | 185 |
| 72142 | Rottweiler | CLEAR | F | 01/05/21 | 09/04/22 | 343 |
| 72166 | Rough Collie | AFFECTED | M | 17/03/20 | 10/04/22 | 754 |
| 72167 | German shepherd | CARRIER | F | 17/09/21 | 10/03/22 | 174 |
| 72169 | German shepherd | CARRIER | F | 30/03/19 | 04/10/22 | 1284 |
| 72173 | Cesky Terrier | CLEAR | F | 26/04/19 | 10/04/22 | 1080 |
| 72184 | White Swiss Shepherd Dog | CLEAR | F | 02/07/21 | 10/04/22 | 282 |
| 72257 | Welsh Corgi Pembroke | AFFECTED | f | 10/03/21 | 07/04/22 | 393 |
| 72322 | German shepherd | CLEAR | M | 23/03/21 | 04/08/22 | 499 |
| 72327 | Dobermann Pinscher | CLEAR | F | 25/12/15 | 13/04/22 | 2301 |
| 72328 | Czechoslovakian wolfdog | AFFECTED | M | 19/03/12 | 12/04/22 | 3676 |
| 72448 | Miniature poodle | CARRIER | F | 23/12/19 | 20/04/22 | 849 |
| 72481 | Bernese Mountain Dog | CLEAR | M | 27/11/20 | 09/04/22 | 498 |
| 72503 | German shepherd | CARRIER | F | 23/07/20 | 20/04/22 | 636 |
| 72526 | Welsh Corgi Pembroke | CLEAR | F | 10/01/22 | 27/04/22 | 107 |
| 72628 | Australian shepherd | CLEAR | F | 02/04/21 | 29/04/22 | 392 |
| 72630 | Dogue de Bordeaux | CLEAR | F | 09/06/21 | 28/04/22 | 323 |

| | | | | | | |
|---|---|---|---|---|---|---|
| 72671 | Welsh Corgi Pembroke | CARRIER | F | 08/02/22 | 02/05/22 | 83 |
| 72674 | White Swiss Shepherd Dog | CLEAR | | | 02/05/22 | |
| 72676 | Welsh Terrier | CLEAR | F | 03/02/18 | 02/05/22 | 1549 |
| 72758 | German shepherd | CARRIER | F | 05/02/18 | 02/05/22 | 1547 |
| 72767 | Bernese Mountain Dog | CLEAR | M | 29/12/18 | 04/05/22 | 1222 |
| 72817 | Bernese Mountain Dog | CARRIER | F | 14/03/21 | 04/05/22 | 416 |
| 72818 | Bernese Mountain Dog | CARRIER | F | 04/04/21 | 04/05/22 | 395 |
| 72823 | Bernese Mountain Dog | CARRIER | M | 16/03/19 | 12/04/22 | 1123 |
| 72971 | Poodle | CLEAR | M | 21/06/21 | 08/05/22 | 321 |
| 72973 | Poodle | CLEAR | F | 17/01/21 | 08/05/22 | 476 |
| 72985 | French Bulldog | CARRIER | F | 09/12/20 | 08/05/22 | 515 |
| 72990 | Australian shepherd | CLEAR | M | 04/07/20 | 10/05/22 | 675 |
| 72991 | English setter | CLEAR | F | 15/11/18 | 11/05/22 | 1273 |
| 72992 | English setter | CLEAR | M | 15/06/15 | 11/05/22 | 2522 |
| 72993 | English setter | CLEAR | M | 15/11/18 | 11/05/22 | 1273 |
| 72998 | German shepherd | CLEAR | M | 13/04/17 | 26/04/22 | 1839 |
| 73101 | German shepherd | CLEAR | M | 07/05/21 | 05/07/22 | 424 |
| 73121 | Miniature poodle | CLEAR | | | 15/05/22 | |
| 73126 | Weimaraner | CLEAR | M | 01/05/21 | 15/05/22 | 379 |
| 73128 | Poodle | CLEAR | | | 15/05/22 | |
| 73129 | Poodle | CLEAR | | | 15/05/22 | |
| 73131 | White Swiss Shepherd Dog | CLEAR | | | 15/05/22 | |
| 73153 | Belgian shepherd Malinois | CLEAR | M | 07/05/18 | 15/05/22 | 1469 |
| 73155 | Poodle | CLEAR | F | 26/10/21 | 15/05/22 | 201 |
| 73183 | Czechoslovakian wolfdog | CLEAR | M | 25/01/20 | 16/05/22 | 842 |
| 73213 | Shiba inu | CLEAR | F | 24/09/21 | 14/05/22 | 232 |
| 73272 | Miniature poodle | CLEAR | F | 06/04/19 | 18/05/22 | 1138 |
| 73308 | French Bulldog | CARRIER | F | 29/04/20 | 09/05/22 | 740 |
| 73366 | Poodle | CLEAR | F | 18/07/17 | 23/05/22 | 1770 |
| 73410 | Miniature poodle | CLEAR | F | 03/09/20 | 23/05/22 | 627 |
| 73415 | Poodle | CLEAR | F | 17/03/20 | 25/05/22 | 799 |
| 73431 | Toy poodle | CLEAR | M | 18/03/20 | 24/05/22 | 797 |
| 73499 | Miniature American shepherd | CLEAR | F | 29/08/21 | 28/05/22 | 272 |
| 73500 | Miniature American shepherd | CLEAR | F | 31/08/21 | 28/05/22 | 270 |

| | | | | | | |
|---|---|---|---|---|---|---|
| 73528 | Australian cattledog | CLEAR | M | 07/02/21 | 29/05/22 | 476 |
| 73547 | Poodle | CLEAR | | | 29/05/22 | |
| 73550 | Poodle | CLEAR | M | 24/04/21 | 29/05/22 | 400 |
| 73551 | Dutch Shepherd Dog | CLEAR | F | 16/10/18 | 29/05/22 | 1321 |
| 73553 | Poodle | CLEAR | | | 29/05/22 | |
| 73570 | German shepherd | CLEAR | M | 26/03/21 | 24/05/22 | 424 |
| 73583 | Welsh Corgi Pembroke | CLEAR | F | 05/04/22 | 19/05/22 | 44 |
| 73584 | Welsh Corgi Pembroke | CARRIER | F | 05/04/22 | 19/05/22 | 44 |
| 73585 | Welsh Corgi Pembroke | CLEAR | F | 05/04/22 | 19/05/22 | 44 |
| 73589 | Czechoslovakian wolfdog | CARRIER | F | 30/11/19 | 28/04/22 | 880 |
| 73599 | German shepherd | CLEAR | F | 07/05/21 | 30/05/22 | 388 |
| 73606 | Bernese Mountain Dog | CLEAR | M | 18/01/21 | 31/05/22 | 498 |
| 73731 | Bernese Mountain Dog | CLEAR | M | 08/05/21 | 04/06/22 | 392 |
| 73753 | Poodle | CLEAR | M | 26/11/20 | 05/06/22 | 556 |
| 73766 | Bernese Mountain Dog | CLEAR | M | 09/02/21 | 16/05/22 | 461 |
| 73800 | Mixed breed | CLEAR | M | 09/03/18 | 03/06/22 | 1547 |
| 73803 | Czechoslovakian wolfdog | CLEAR | M | 15/02/14 | 25/05/22 | 3021 |
| 73819 | Labrador retriever | CLEAR | M | 01/07/12 | 03/06/22 | 3624 |
| 73823 | Bernese Mountain Dog | CLEAR | F | 09/02/21 | 04/06/22 | 480 |
| 73860 | Rough Collie | CLEAR | M | 12/05/21 | 08/06/22 | 392 |
| 73862 | astore della Lessinia e del Lagor | CLEAR | M | 16/09/21 | 06/06/22 | 263 |
| 73866 | Rough Collie | CARRIER | F | 28/05/22 | 08/06/22 | 11 |
| 73897 | Australian shepherd | CLEAR | F | 01/12/19 | 08/06/22 | 920 |
| 73917 | Australian cattledog | CLEAR | M | 18/08/17 | 07/06/22 | 1754 |
| 73918 | Australian cattledog | CLEAR | F | 15/06/20 | 28/05/22 | 712 |
| 73978 | Welsh Corgi Pembroke | AFFECTED | F | 10/03/21 | 10/06/22 | 457 |
| 74038 | Australian shepherd | CLEAR | M | 12/04/20 | 06/11/22 | 938 |
| 74039 | Australian shepherd | CLEAR | F | 12/04/20 | 11/06/22 | 790 |
| 74042 | White Swiss Shepherd Dog | CLEAR | M | 23/03/20 | 13/06/22 | 812 |
| 74046 | German shepherd | AFFECTED | M | 12/09/14 | 15/06/22 | 2833 |
| 74067 | Poodle | CLEAR | F | 25/05/21 | 18/06/22 | 389 |
| 74073 | Poodle | CLEAR | F | 17/11/21 | 18/06/22 | 213 |
| 74114 | Cavalier King Charles Spaniel | CARRIER | F | 02/12/20 | 19/06/22 | 564 |
| 74140 | German shepherd | CARRIER | F | 30/10/20 | 23/05/22 | 570 |

| | | | | | | |
|---|---|---|---|---|---|---|
| 74146 | German shepherd | CLEAR | F | 24/06/17 | 20/06/22 | 1822 |
| 74150 | Australian shepherd | CLEAR | F | 15/07/21 | 31/05/22 | 320 |
| 74152 | Australian shepherd | CLEAR | F | 02/05/21 | 31/05/22 | 394 |
| 74153 | Australian shepherd | CLEAR | M | 29/11/20 | 31/05/22 | 548 |
| 74265 | Pinscher | CLEAR | F | 10/02/18 | 21/06/22 | 1592 |
| 74278 | German shepherd | CLEAR | M | 14/03/22 | 28/06/22 | 106 |
| 74284 | Australian shepherd | CARRIER | M | 15/07/18 | 28/06/22 | 1444 |
| 74285 | Australian shepherd | CARRIER | F | 19/07/21 | 28/06/22 | 344 |
| 74290 | German shepherd | CLEAR | M | 10/12/17 | 27/06/22 | 1660 |
| 74366 | White Swiss Shepherd Dog | CLEAR | F | 08/02/21 | 28/06/22 | 505 |
| 74385 | German shepherd | AFFECTED | M | 07/02/21 | 20/06/22 | 498 |
| 74438 | Kerry blue terrier | CLEAR | M | 17/01/20 | 01/07/22 | 896 |
| 74455 | Poodle | CLEAR | M | 17/06/21 | 02/07/22 | 380 |
| 74456 | Miniature poodle | CARRIER | F | 24/04/21 | 02/07/22 | 434 |
| 74457 | Poodle | CLEAR | F | 20/05/21 | 02/07/22 | 408 |
| 74458 | Poodle | CLEAR | F | 22/05/21 | 02/07/22 | 406 |
| 74459 | Miniature poodle | CLEAR | F | 24/04/21 | 02/07/22 | 434 |
| 74478 | Miniature American shepherd | CLEAR | F | 05/01/21 | 02/07/22 | 543 |
| 74479 | Miniature American shepherd | CLEAR | F | 27/04/21 | 02/07/22 | 431 |
| 74532 | Hovawart | CLEAR | M | 02/04/21 | 21/06/22 | 445 |
| 74585 | White Swiss Shepherd Dog | CLEAR | F | 06/06/21 | 07/07/22 | 396 |
| 74594 | Toy poodle | CLEAR | F | 21/04/21 | 07/07/22 | 442 |
| 74595 | Toy poodle | CLEAR | F | 11/02/22 | 07/07/22 | 146 |
| 74596 | Toy poodle | CLEAR | M | 01/10/20 | 07/07/22 | 644 |
| 74625 | White Swiss Shepherd Dog | CLEAR | F | 31/10/20 | 08/07/22 | 615 |
| 74626 | Belgian shepherd Tervueren | CLEAR | M | 11/02/21 | 11/07/22 | 515 |
| 74628 | German shepherd | AFFECTED | M | 26/12/15 | 11/07/22 | 2389 |
| 74651 | Cane corso | CLEAR | M | 18/10/14 | 11/07/22 | 2823 |
| 74691 | French Bulldog | CLEAR | F | 23/06/21 | 13/07/22 | 385 |
| 74694 | Australian shepherd | CLEAR | F | 06/01/20 | 12/07/22 | 918 |
| 74720 | Welsh Corgi Pembroke | CARRIER | F | 13/10/21 | 13/07/22 | 273 |
| 74878 | Czechoslovakian wolfdog | CLEAR | M | 09/04/21 | 19/07/22 | 466 |
| 74894 | Mixed breed | AFFECTED | M | 03/11/11 | 25/07/22 | 3917 |
| 74923 | White Swiss Shepherd Dog | CARRIER | M | 15/01/21 | 25/07/22 | 556 |

| | | | | | | |
|---|---|---|---|---|---|---|
| 74924 | Welsh Corgi Pembroke | CLEAR | F | 11/04/22 | 25/07/22 | 105 |
| 74936 | Bernese Mountain Dog | CLEAR | M | 16/11/20 | 19/07/22 | 610 |
| 74952 | Apuan Alps' sheperd | CARRIER | M | 16/06/22 | 23/07/22 | 37 |
| 74969 | German shepherd | CLEAR | M | 08/07/21 | 26/07/22 | 383 |
| 75013 | Weimaraner | CLEAR | F | 15/06/21 | 29/07/22 | 409 |
| 75032 | Poodle | CLEAR | | | 29/07/22 | |
| 75080 | Rough Collie | CARRIER | F | 08/08/20 | 29/07/22 | 720 |
| 75143 | Poodle | CARRIER | M | 02/08/21 | 02/08/22 | 365 |
| 75144 | Poodle | CLEAR | M | 11/02/21 | 02/08/22 | 537 |
| 75145 | Poodle | CLEAR | M | 11/10/21 | 02/08/22 | 295 |
| 75175 | Hovawart | CLEAR | M | 16/07/21 | 01/08/22 | 381 |
| 75181 | Cavalier King Charles Spaniel | AFFECTED | F | 15/12/21 | 01/08/22 | 229 |
| 75229 | German shepherd | CARRIER | M | 26/06/21 | 25/07/22 | 394 |
| 75230 | German shepherd | CLEAR | F | 27/11/20 | 08/04/22 | 497 |
| 75279 | Australian shepherd | CLEAR | M | 20/11/20 | 09/08/22 | 627 |
| 75280 | Australian shepherd | CLEAR | F | 24/02/21 | 09/08/22 | 531 |
| 75288 | German shepherd | CARRIER | F | 15/09/20 | 07/08/22 | 691 |
| 75312 | German shepherd | CLEAR | M | 20/10/21 | 10/08/22 | 294 |
| 75316 | Shih tzu | CARRIER | F | 28/12/19 | 10/08/22 | 956 |
| 75317 | Shih tzu | CLEAR | M | 09/06/19 | 10/08/22 | 1158 |
| 75358 | French Bulldog | CARRIER | F | 01/06/21 | 19/08/22 | 444 |
| 75443 | Welsh Corgi Pembroke | CLEAR | M | 03/12/20 | 22/08/22 | 627 |
| 75449 | Wire Fox terrier | AFFECTED | M | 20/03/16 | 22/08/22 | 2346 |
| 75455 | Welsh Corgi Pembroke | AFFECTED | | | 22/08/22 | |
| 75457 | American Staffordshire Terrier | CLEAR | M | 14/09/20 | 22/08/22 | 707 |
| 75477 | Dachshund | CLEAR | F | 28/08/21 | 08/09/22 | 376 |
| 75481 | Czechoslovakian wolfdog | CLEAR | F | 20/11/20 | 24/08/22 | 642 |
| 75482 | Cavalier King Charles Spaniel | CARRIER | F | 10/05/21 | 24/08/22 | 471 |
| 75507 | German shepherd | CARRIER | F | 18/08/11 | 26/08/22 | 4026 |
| 75528 | Belgian shepherd Malinois | CLEAR | F | 24/02/21 | 25/08/22 | 547 |
| 75534 | Hovawart | CLEAR | M | 25/06/16 | 22/08/22 | 2249 |
| 75539 | Rough Collie | CLEAR | M | 23/06/22 | 26/08/22 | 64 |
| 75621 | White Swiss Shepherd Dog | CLEAR | F | 18/11/21 | 28/08/22 | 283 |
| 75636 | Cavalier King Charles Spaniel | CARRIER | F | 31/10/21 | 28/08/22 | 301 |

| | | | | | | |
|---|---|---|---|---|---|---|
| 75645 | Weimaraner | CLEAR | F | 17/10/21 | 28/08/22 | 315 |
| 75646 | Weimaraner | CLEAR | F | 05/01/20 | 28/08/22 | 966 |
| 75660 | laremmano-Abruzzese Sheepdo | CLEAR | F | 14/02/15 | 27/08/22 | 2751 |
| 75674 | German shepherd | AFFECTED | F | 21/03/21 | 30/08/22 | 527 |
| 75678 | Shetland shepherd | CLEAR | M | 18/01/17 | 30/08/22 | 2050 |
| 75682 | Rough Collie | CARRIER | F | 14/08/21 | 29/08/22 | 380 |
| 75693 | French Bulldog | CARRIER | M | 10/06/21 | 30/08/22 | 446 |
| 75743 | Shetland Sheepdog | CLEAR | F | 11/04/21 | 30/08/22 | 506 |
| 75772 | Poodle | CLEAR | M | 10/05/19 | 09/03/22 | 1034 |
| 75794 | Rough Collie | CLEAR | M | 31/03/19 | 06/09/22 | 1255 |
| 75798 | Australian shepherd | CLEAR | M | 10/11/20 | 06/09/22 | 665 |
| 75848 | Cavalier King Charles Spaniel | AFFECTED | | | 06/09/22 | |
| 75858 | Bernese Mountain Dog | CARRIER | F | 23/04/19 | 09/07/22 | 1173 |
| 75898 | German shepherd | CLEAR | M | 08/08/10 | 06/09/22 | 4412 |
| 75962 | Czechoslovakian wolfdog | CLEAR | M | 01/08/21 | 12/09/22 | 407 |
| 76026 | Shetland shepherd | CLEAR | M | 16/05/22 | 17/09/22 | 124 |
| 76047 | Toy poodle | CLEAR | F | 29/10/19 | 17/09/22 | 1054 |
| 76061 | Welsh Corgi Cardigan | CLEAR | M | 22/11/20 | 17/09/22 | 664 |
| 76111 | Welsh Terrier | CLEAR | | | 17/09/22 | |
| 76115 | Czechoslovakian wolfdog | CLEAR | M | 02/01/22 | 17/09/22 | 258 |
| 76236 | Australian shepherd | CLEAR | M | 25/02/21 | 19/09/22 | 571 |
| 76237 | Australian shepherd | CARRIER | F | 18/08/21 | 19/09/22 | 397 |
| 76265 | German shepherd | CARRIER | M | 08/04/14 | 16/09/22 | 3083 |
| 76266 | German shepherd | CARRIER | M | 15/12/14 | 09/09/22 | 2825 |
| 76313 | German shepherd | CLEAR | F | 20/08/20 | 21/09/22 | 762 |
| 76317 | Shetland shepherd | CARRIER | M | 23/03/22 | 22/09/22 | 183 |
| 76332 | Cavalier King Charles Spaniel | AFFECTED | M | 14/02/22 | 24/09/22 | 222 |
| 76409 | Miniature poodle | CLEAR | | | 24/09/22 | |
| 76431 | Poodle | CLEAR | | | 24/09/22 | |
| 76490 | German shepherd | CLEAR | M | 01/10/14 | 26/09/22 | 2917 |
| 76504 | Chihuahua | CLEAR | M | 04/02/20 | 27/09/22 | 966 |
| 76505 | Deutscher Zwergspitz | CLEAR | M | 28/12/18 | 27/09/22 | 1369 |
| 76506 | Maltese | CLEAR | M | 10/04/20 | 27/09/22 | 900 |
| 76591 | Czechoslovakian wolfdog | CLEAR | M | 06/06/21 | 21/09/22 | 472 |

| | | | | | | |
|---|---|---|---|---|---|---|
| 76595 | White Swiss Shepherd Dog | CLEAR | M | 18/09/21 | 28/09/22 | 375 |
| 76611 | German shepherd | CLEAR | F | 27/05/18 | 29/09/22 | 1586 |
| 76612 | German shepherd | CLEAR | M | 07/08/17 | 29/09/22 | 1879 |
| 76618 | astore della Lessinia e del Lagor | CLEAR | F | 01/10/20 | 28/09/22 | 727 |
| 76619 | astore della Lessinia e del Lagor | CLEAR | M | 17/12/17 | 28/09/22 | 1746 |
| 76649 | Cane corso | CLEAR | M | 24/09/20 | 04/10/22 | 740 |
| 76665 | German shepherd | CLEAR | M | 23/04/22 | 03/10/22 | 163 |
| 76684 | Poodle | CLEAR | M | 26/11/20 | 06/10/22 | 679 |
| 76685 | German shepherd | CLEAR | F | 08/04/21 | 05/10/22 | 545 |
| 76686 | German shepherd | CLEAR | F | 23/09/21 | 05/10/22 | 377 |
| 76687 | German shepherd | CLEAR | F | 11/08/21 | 05/10/22 | 420 |
| 76688 | German shepherd | CLEAR | F | 28/08/21 | 05/10/22 | 403 |
| 76697 | Shetland shepherd | CLEAR | F | 21/09/21 | 06/10/22 | 380 |
| 76716 | Rottweiler | CLEAR | | | | |
| 76720 | Cavalier King Charles Spaniel | CARRIER | F | 21/05/21 | 26/09/22 | 493 |
| 76772 | Czechoslovakian wolfdog | CLEAR | | | | |
| 76775 | Welsh Corgi Pembroke | AFFECTED | F | 22/09/21 | 10/10/22 | 383 |
| 76776 | Welsh Corgi Pembroke | CARRIER | M | 01/11/21 | 10/10/22 | 343 |
| 76784 | Czechoslovakian wolfdog | CLEAR | F | 08/08/22 | 08/10/22 | 61 |
| 76794 | Poodle | CLEAR | F | 06/03/19 | 06/10/22 | 1310 |
| 76806 | French Bulldog | CARRIER | F | 28/11/20 | 07/10/22 | 678 |
| 76834 | French Bulldog | CLEAR | F | 21/09/20 | 08/10/22 | 747 |
| 76840 | German shepherd | AFFECTED | M | 22/03/13 | 10/10/22 | 3489 |
| 76845 | Bernese Mountain Dog | CLEAR | F | 27/03/21 | 11/10/22 | 563 |
| 76846 | French Bulldog | CLEAR | F | 07/07/21 | 06/10/22 | 456 |
| 76847 | French Bulldog | CARRIER | F | 11/10/21 | 06/10/22 | 360 |
| 76904 | Weimaraner | CLEAR | F | 28/05/21 | 14/10/22 | 504 |
| 76912 | Australian shepherd | CLEAR | M | 27/01/21 | 11/10/22 | 622 |
| 76939 | Bernese Mountain Dog | CLEAR | M | 07/05/22 | 10/10/22 | 156 |
| 76953 | Pumi | CLEAR | M | 29/03/20 | 15/10/22 | 930 |
| 76955 | Wire Fox terrier | AFFECTED | F | 19/07/21 | 15/10/22 | 453 |
| 76989 | Poodle | CLEAR | F | 16/08/21 | 15/10/22 | 425 |
| 77032 | Poodle | CLEAR | F | 09/02/21 | 13/10/22 | 611 |
| 77033 | Poodle | CARRIER | M | 26/08/21 | 13/10/22 | 413 |

| | | | | | | |
|---|---|---|---|---|---|---|
| 77045 | Poodle | CLEAR | F | 26/12/17 | 17/10/22 | 1756 |
| 77054 | astore della Lessinia e del Lagorai | | F | 29/05/21 | 19/10/22 | 508 |
| 77055 | Belgian shepherd Malinois | CLEAR | F | 29/08/21 | 20/10/22 | 417 |
| 77063 | Czechoslovakian wolfdog | CLEAR | M | 10/03/20 | 14/10/22 | 948 |
| 77070 | German shepherd | CLEAR | F | 12/01/17 | 17/10/22 | 2104 |
| 77103 | Toy poodle | CLEAR | F | 20/11/20 | 15/10/22 | 694 |
| 77154 | German shepherd | AFFECTED | F | 01/01/14 | 25/10/22 | 3219 |
| 77181 | German shepherd | CLEAR | M | 17/09/21 | 20/10/22 | 398 |
| 77182 | German shepherd | CLEAR | F | 26/01/20 | 20/10/22 | 998 |
| 77183 | Bobtail | CLEAR | F | 07/12/18 | 25/10/22 | 1418 |
| 77196 | French Bulldog | CLEAR | F | 15/11/21 | 24/11/22 | 374 |
| 77241 | Västgötaspets | CLEAR | F | 07/10/21 | 29/10/22 | 387 |
| 77253 | Beauceron | | F | 09/07/21 | 29/10/22 | 477 |
| 77257 | Australian shepherd | CARRIER | M | 12/04/22 | 29/10/22 | 200 |
| 77267 | American akita | CLEAR | F | 06/12/21 | 29/10/22 | 327 |
| 77290 | Poodle | CLEAR | F | 04/04/20 | 30/10/22 | 939 |
| 77292 | Poodle | CLEAR | M | 24/10/21 | 30/10/22 | 371 |
| 77306 | Poodle | CLEAR | F | 13/03/21 | 30/10/22 | 596 |
| 77314 | German shepherd | CLEAR | M | 01/05/21 | 30/10/22 | 547 |
| 77315 | Australian shepherd | CLEAR | F | 07/09/20 | 30/10/22 | 783 |
| 77316 | Toy poodle | CLEAR | F | 04/08/21 | 30/10/22 | 452 |
| 77331 | German shepherd | CARRIER | F | 28/02/21 | 26/10/22 | 605 |
| 77333 | Shih tzu | CARRIER | M | 06/09/19 | 28/10/22 | 1148 |
| 77334 | Shih tzu | CLEAR | F | 05/04/20 | 28/10/22 | 936 |
| 77335 | Lhasa Apso | CARRIER | F | 15/10/20 | 28/10/22 | 743 |
| 77368 | Poodle | CLEAR | F | 14/08/21 | 01/11/22 | 444 |
| 77369 | Poodle | CLEAR | F | 27/11/21 | 01/11/22 | 339 |
| 77370 | Kai | CLEAR | M | 17/06/19 | 01/11/22 | 1233 |
| 77372 | Barzoi | CARRIER | F | 29/11/21 | 01/11/22 | 337 |
| 77421 | Cavalier King Charles Spaniel | CARRIER | F | 18/10/19 | 27/10/22 | 1105 |
| 77446 | Lapinkoira | CLEAR | M | 21/09/18 | 02/11/22 | 1503 |
| 77475 | Czechoslovakian wolfdog | CLEAR | M | 03/10/21 | 11/06/22 | 251 |
| 77535 | Poodle | CLEAR | F | 20/03/21 | 05/11/22 | 595 |
| 77538 | Miniature poodle | CLEAR | M | 23/06/21 | 05/11/22 | 500 |

| | | | | | | |
|---|---|---|---|---|---|---|
| 77617 | Australian shepherd | CLEAR | F | 28/06/21 | 07/11/22 | 497 |
| 77618 | Cane corso | CLEAR | M | | 07/11/22 | |
| 77626 | Czechoslovakian wolfdog | CARRIER | M | 06/02/18 | 05/11/22 | 1733 |
| 77644 | Cavalier King Charles Spaniel | CARRIER | M | 24/12/21 | 09/11/22 | 320 |
| 77645 | Cavalier King Charles Spaniel | CARRIER | F | 15/02/21 | 09/11/22 | 632 |
| 77646 | Toy poodle | CLEAR | F | 11/06/18 | 09/11/22 | 1612 |
| 77647 | Toy poodle | CLEAR | M | 19/05/19 | 09/11/22 | 1270 |
| 77661 | White Swiss Shepherd Dog | CARRIER | M | 29/12/18 | 09/11/22 | 1411 |
| 77680 | Welsh Corgi Pembroke | CARRIER | F | 13/01/21 | 08/11/22 | 664 |
| 77680 | Welsh Corgi Pembroke | CARRIER | F | 13/01/21 | 08/11/22 | 664 |
| 77695 | Bernese Mountain Dog | CLEAR | M | 21/04/20 | 11/11/22 | 934 |
| 77699 | Rhodesian Ridgeback | CLEAR | M | 13/01/20 | 08/11/22 | 1030 |
| 77725 | Poodle | CLEAR | F | 16/12/21 | 14/11/22 | 333 |
| 77726 | Poodle | CLEAR | F | 16/12/21 | 14/11/22 | 333 |
| 77792 | Bernese Mountain Dog | CLEAR | M | | 09/11/22 | |
| 77808 | Czechoslovakian wolfdog | CLEAR | M | 16/09/21 | 15/11/22 | 425 |
| 77813 | Australian shepherd | CLEAR | F | 05/09/21 | 12/11/22 | 433 |
| 77930 | Czechoslovakian wolfdog | CARRIER | M | 16/12/10 | 22/11/22 | 4359 |
| 77947 | German shepherd | CLEAR | F | 03/11/18 | 28/11/22 | 1486 |
| 78013 | Australian shepherd | CLEAR | M | 22/09/19 | 24/11/22 | 1159 |
| 78015 | German shepherd | CLEAR | M | 28/09/16 | 28/11/22 | 2252 |
| 78031 | White Swiss Shepherd Dog | CLEAR | F | 24/12/20 | 01/12/22 | 707 |
| 78050 | Czechoslovakian wolfdog | CLEAR | F | 05/12/20 | 30/11/22 | 725 |
| 78081 | Australian shepherd | CLEAR | F | 13/11/21 | 03/12/22 | 385 |
| 78091 | Belgian shepherd Malinois | CLEAR | M | | 05/12/22 | |
| 78093 | Belgian shepherd Tervueren | CLEAR | M | 09/02/22 | 05/12/22 | 299 |
| 78122 | French Bulldog | CLEAR | M | 03/01/22 | 06/12/22 | 337 |
| 78134 | astore della Lessinia e del Lagor | CLEAR | F | 21/05/21 | 28/11/22 | 556 |
| 78142 | Poodle | CLEAR | F | 06/04/21 | 06/12/22 | 609 |
| 78143 | Australian shepherd | CLEAR | M | 08/11/21 | 17/11/22 | 374 |
| 78149 | Czechoslovakian wolfdog | CLEAR | F | 19/09/20 | 30/11/22 | 802 |
| 78156 | German shepherd | CLEAR | F | 12/07/21 | 26/10/22 | 471 |
| 78177 | Miniature poodle | CLEAR | M | 06/01/19 | 13/12/22 | 1437 |
| 78178 | Miniature poodle | CLEAR | F | 25/02/20 | 13/12/22 | 1022 |

| | | | | | | |
|---|---|---|---|---|---|---|
| 78183 | German shepherd | CARRIER | F | 18/02/09 | 06/12/22 | 5039 |
| 78193 | Australian shepherd | CARRIER | F | 18/06/21 | 18/11/22 | 518 |
| 78223 | Lhasa Apso | CLEAR | M | 12/03/19 | 14/12/22 | 1373 |
| 78224 | Tibetan spaniel | CLEAR | F | 25/03/21 | 14/12/22 | 629 |
| 78225 | Shih tzu | CLEAR | F | 07/03/17 | 14/12/22 | 2108 |
| 78272 | Czechoslovakian wolfdog | CLEAR | M | 03/10/21 | 15/12/22 | 438 |
| 78377 | Kai | CLEAR | F | 22/02/20 | 18/12/22 | 1030 |
| 78447 | Welsh Corgi Pembroke | CARRIER | F | 29/05/21 | 15/12/22 | 565 |
| 78622 | Belgian shepherd Malinois | CLEAR | F | 26/01/21 | 14/12/22 | 687 |
| 78623 | Belgian shepherd Malinois | CLEAR | F | 02/08/17 | 14/12/22 | 1960 |
| 78633 | French Bulldog | CLEAR | M | 23/09/22 | 07/12/22 | 75 |
| 78663 | Dobermann Pinscher | CLEAR | M | 02/07/21 | 20/12/22 | 536 |
| 78674 | Welsh Corgi Pembroke | CARRIER | M | 16/08/22 | 15/12/22 | 121 |
| 78757 | German shepherd | CLEAR | M | 03/04/17 | 21/12/22 | 2088 |
| 78770 | Australian shepherd | CLEAR | F | 12/04/20 | 21/12/22 | 983 |
| 78792 | Australian Kelpie | AFFECTED | M | 16/02/12 | 27/12/22 | 3967 |
| 78793 | Weimaraner | CLEAR | M | 05/01/20 | 28/12/22 | 1088 |
| 78804 | German shepherd | CARRIER | F | 05/12/21 | 19/12/22 | 379 |
| 78817 | Welsh Corgi Cardigan | CLEAR | F | 02/03/19 | 01/03/23 | 1460 |
| 78824 | White Swiss Shepherd Dog | CLEAR | F | 07/06/21 | 24/12/22 | 565 |
| 78841 | German shepherd | CARRIER | F | 07/09/18 | 13/12/22 | 1558 |
| 78846 | Dobermann Pinscher | CLEAR | M | 01/09/21 | 01/03/23 | 546 |
| 78864 | Barzoi | CLEAR | M | 29/11/21 | 01/10/23 | 671 |
| 78892 | Australian shepherd | CLEAR | M | 13/10/22 | 09/01/23 | 88 |
| 78906 | Australian shepherd | CARRIER | F | 02/05/22 | 09/01/23 | 252 |
| 78941 | Czechoslovakian wolfdog | AFFECTED | F | 02/10/14 | 23/12/22 | 3004 |
| 78951 | Bernese Mountain Dog | CLEAR | F | 06/02/22 | 10/01/23 | 338 |
| 78952 | Bernese Mountain Dog | CLEAR | F | 18/11/21 | 10/01/23 | 418 |
| 78959 | Weimaraner | CLEAR | F | 19/07/20 | 21/12/22 | 885 |
| 78970 | Bernese Mountain Dog | CARRIER | M | 18/09/19 | 11/01/23 | 1211 |
| 78971 | Czechoslovakian wolfdog | CLEAR | F | 02/11/20 | 10/01/23 | 799 |
| 78986 | Weimaraner | CLEAR | M | 19/10/20 | 16/01/23 | 819 |
| 78989 | Welsh Corgi Pembroke | AFFECTED | F | 11/12/20 | 11/01/23 | 761 |
| 78997 | Toy poodle | CLEAR | F | 14/04/21 | 13/01/23 | 639 |

| | | | | | | |
|---|---|---|---|---|---|---|
| 79003 | Toy poodle | CLEAR | F | 10/04/20 | 16/01/23 | 1011 |
| 79004 | Toy poodle | CLEAR | F | 10/03/18 | 16/01/23 | 1773 |
| 79008 | Lhasa Apso | CLEAR | M | 12/10/16 | 16/01/23 | 2287 |
| 79009 | Lhasa Apso | CLEAR | F | 25/04/21 | 16/01/23 | 631 |
| 79020 | Poodle | CLEAR | F | 21/09/19 | 16/01/23 | 1213 |
| 79044 | Australian shepherd | CLEAR | M | 03/02/17 | 16/01/23 | 2173 |
| 79067 | German shepherd | CARRIER | F | 17/03/22 | 16/01/23 | 305 |
| 79232 | German shepherd | CARRIER | M | 01/01/13 | 23/01/23 | 3674 |
| 79235 | German shepherd | CLEAR | F | 25/10/18 | 23/01/23 | 1551 |
| 79237 | Czechoslovakian wolfdog | CLEAR | f | 24/11/21 | 14/01/23 | 416 |
| 79272 | German shepherd | CLEAR | F | 12/02/17 | 14/01/23 | 2162 |
| 79275 | Czechoslovakian wolfdog | CLEAR | F | 02/01/22 | 25/01/23 | 388 |
| 79276 | Czechoslovakian wolfdog | CARRIER | M | 21/10/21 | 25/01/23 | 461 |
| 79295 | German shepherd | CLEAR | M | 17/01/22 | 28/01/23 | 376 |
| 79303 | Czechoslovakian wolfdog | CLEAR | M | 04/12/22 | 24/01/23 | 51 |
| 79304 | Czechoslovakian wolfdog | CLEAR | M | 04/12/22 | 24/01/23 | 51 |
| 79306 | German shepherd | AFFECTED | F | 11/10/20 | 31/01/23 | 842 |
| 79353 | Rough Collie | CARRIER | F | 28/03/22 | 27/01/23 | 305 |
| 79465 | Belgian shepherd Malinois | CLEAR | F | 12/08/20 | 30/01/23 | 901 |
| 79492 | astore della Lessinia e del Lagor | CLEAR | F | 21/05/21 | 02/02/23 | 622 |
| 79496 | Bernese Mountain Dog | CLEAR | F | 03/07/21 | 01/02/23 | 578 |
| 79497 | Bernese Mountain Dog | CLEAR | F | 16/05/21 | 01/02/23 | 626 |
| 79543 | Toy poodle | CLEAR | M | 28/03/21 | 02/02/23 | 676 |
| 79593 | Perro sin pelo del Peru | CARRIER | M | 22/03/18 | 24/01/23 | 1769 |
| 79609 | Australian shepherd | AFFECTED | F | 24/08/11 | 06/02/23 | 4184 |
| 79619 | German shepherd | CARRIER | M | 28/11/21 | 31/01/23 | 429 |
| 79621 | Toy poodle | CLEAR | F | 20/03/21 | 07/02/23 | 689 |
| 79658 | French Bulldog | CARRIER | M | 30/01/22 | 07/02/23 | 373 |
| 79664 | Toy poodle | CLEAR | M | 15/09/21 | 08/02/23 | 511 |
| 79745 | Rough Collie | CLEAR | M | 26/08/21 | 12/02/23 | 535 |
| 79754 | Rough Collie | CLEAR | F | 04/06/22 | 12/02/23 | 253 |
| 79765 | Tibetan spaniel | CLEAR | M | 31/07/21 | 10/02/23 | 559 |
| 79766 | Tibetan spaniel | CLEAR | F | 10/08/21 | 10/02/23 | 549 |
| 79838 | Australian shepherd | CLEAR | F | 20/08/20 | 11/01/23 | 874 |

| | | | | | | |
|---|---|---|---|---|---|---|
| 79846 | Border collie | CLEAR | F | 28/05/21 | 15/02/23 | 628 |
| 79847 | Czechoslovakian wolfdog | CLEAR | F | 04/12/22 | 09/02/23 | 67 |
| 79848 | Czechoslovakian wolfdog | CLEAR | F | 04/12/22 | 09/02/23 | 67 |
| 79849 | Czechoslovakian wolfdog | CLEAR | F | 04/12/22 | 09/02/23 | 67 |
| 79871 | Bernese Mountain Dog | CLEAR | F | 05/01/21 | 10/02/23 | 766 |
| 79878 | Cavalier King Charles Spaniel | AFFECTED | M | 29/09/21 | 20/02/23 | 509 |
| 79882 | German shepherd | CARRIER | M | 02/07/13 | 16/02/23 | 3516 |
| 79883 | German shepherd | CLEAR | M | 07/11/21 | 15/02/23 | 465 |
| 79901 | Miniature American shepherd | CLEAR | M | 27/11/22 | 16/02/23 | 81 |
| 79945 | Australian shepherd | CLEAR | F | 01/05/21 | 17/02/23 | 657 |
| 79948 | Border collie | CLEAR | M | 03/03/21 | 18/02/23 | 717 |
| 79952 | French Bulldog | CLEAR | M | 25/11/21 | 18/02/23 | 450 |
| 79982 | French Bulldog | CLEAR | F | 25/11/21 | 19/02/23 | 451 |
| 80085 | Maltese | CLEAR | F | 08/09/21 | 26/02/23 | 536 |
| 80098 | Goldendoodle | CLEAR | F | 04/04/21 | 21/02/23 | 688 |
| 80114 | German shepherd | AFFECTED | M | 15/10/22 | 24/02/23 | 132 |
| 80116 | French Bulldog | CLEAR | M | 18/04/22 | 23/02/23 | 311 |
| 80123 | Toy poodle | CLEAR | F | 28/09/21 | 24/02/23 | 514 |
| 80141 | Poodle | CLEAR | F | 12/11/22 | 21/02/23 | 101 |
| 80176 | Mixed breed | AFFECTED | F | 01/07/11 | 24/02/23 | 4256 |
| 80234 | German shepherd | CLEAR | F | 07/02/22 | 01/03/23 | 387 |
| 80245 | German shepherd | AFFECTED | M | 01/01/17 | 28/02/23 | 2249 |
| 80251 | German shepherd | CLEAR | M | 10/02/21 | 03/03/23 | 751 |
| 80253 | German shepherd | CLEAR | M | 17/02/22 | 28/02/22 | 11 |
| 80270 | Australian shepherd | CLEAR | F | 12/05/21 | 21/02/23 | 650 |
| 80325 | German shepherd | CLEAR | M | 22/12/17 | 04/03/23 | 1898 |
| 80383 | Bernese Mountain Dog | CLEAR | M | 14/11/20 | 08/03/23 | 844 |
| 80387 | German shepherd | CLEAR | M | 03/11/20 | 08/03/23 | 855 |
| 80388 | Belgian shepherd Malinois | CLEAR | F | 30/08/22 | 08/03/23 | 190 |
| 80431 | German shepherd | AFFECTED | F | 23/09/15 | 03/08/23 | 2871 |
| 80449 | astore della Lessinia e del Lagor | CLEAR | F | 06/01/21 | 02/03/23 | 785 |
| 80453 | Australian shepherd | CLEAR | F | 18/03/21 | 13/03/23 | 725 |
| 80454 | Australian shepherd | CLEAR | F | 07/04/21 | 13/03/23 | 705 |
| 80516 | Bernese Mountain Dog | CARRIER | M | 31/01/19 | 11/03/23 | 1500 |

| | | | | | | | |
|---|---|---|---|---|---|---|---|
| 80520 | Poodle | CLEAR | M | 22/05/21 | 11/03/23 | 658 |
| 80522 | Australian shepherd | CLEAR | F | 29/02/20 | 03/09/23 | 1282 |
| 80523 | Australian shepherd | CLEAR | F | 29/02/20 | 07/03/23 | 1102 |
| 80535 | Poodle | CLEAR | F | 26/11/21 | 14/03/23 | 473 |
| 80594 | Australian shepherd | CLEAR | F | 17/04/17 | 15/03/23 | 2158 |
| 80601 | Australian shepherd | CLEAR | M | 02/05/21 | 14/03/23 | 681 |
| 80621 | French Bulldog | CLEAR | F | 10/12/21 | 10/02/23 | 427 |
| 80630 | German shepherd | CARRIER | M | 20/05/21 | 10/03/23 | 659 |
| 80634 | Golden retriever | CLEAR | M | 02/02/22 | 15/03/23 | 406 |
| 80647 | Belgian shepherd Malinois | CLEAR | M | 02/10/22 | 14/03/23 | 163 |
| 80684 | Rough Collie | CARRIER | F | 08/08/20 | 20/03/23 | 954 |
| 80703 | German shepherd | AFFECTED | M | 23/10/14 | 21/03/22 | 2706 |
| 80715 | German shepherd | CARRIER | F | 25/04/19 | 10/03/23 | 1415 |
| 80717 | Bernese Mountain Dog | CLEAR | M | 19/08/21 | 20/03/23 | 578 |
| 80727 | Rough Collie | CLEAR | F | 15/01/21 | 20/03/23 | 794 |
| 80751 | German shepherd | CARRIER | M | 16/09/22 | 20/03/23 | 185 |
| 80828 | Chien de Saint-Hubert | CARRIER | F | 27/01/21 | 25/03/23 | 787 |
| 80829 | Miniature poodle | CLEAR | F | 18/05/21 | 25/03/23 | 676 |
| 80859 | German shepherd | CARRIER | M | 05/12/21 | 23/03/23 | 473 |
| 80872 | Rhodesian Ridgeback | CLEAR | M | 15/06/15 | 28/03/22 | 2478 |
| 80905 | Bernese Mountain Dog | CLEAR | M | 03/02/21 | 27/03/23 | 782 |
| 80929 | Bernese Mountain Dog | CLEAR | M | 06/01/21 | 27/03/23 | 810 |
| 80948 | French Bulldog | CLEAR | M | 01/01/21 | 28/03/23 | 816 |
| 80972 | Cavalier King Charles Spaniel | CLEAR | F | 09/11/21 | 01/04/23 | 508 |
| 80973 | Cavalier King Charles Spaniel | CLEAR | M | 09/11/21 | 01/04/23 | 508 |
| 80984 | Black pug dog | CLEAR | F | 08/09/22 | 02/04/23 | 206 |
| 81124 | Poodle | CLEAR | M | 05/06/21 | 05/04/23 | 669 |
| 81223 | German shepherd | AFFECTED | F | 30/09/14 | 12/04/23 | 3116 |
| 81229 | Chien de Saint-Hubert | CARRIER | F | 05/02/21 | 11/04/23 | 795 |
| 81232 | Border collie | CLEAR | M | 11/09/20 | 12/04/23 | 943 |
| 81233 | Maltese | CLEAR | F | 08/09/21 | 17/04/23 | 586 |
| 81241 | Apuan Alps' sheperd | CARRIER | M | 02/07/21 | 11/04/23 | 648 |
| 81285 | Czechoslovakian wolfdog | CLEAR | F | 01/01/22 | 17/04/23 | 471 |
| 81290 | Bernese Mountain Dog | CARRIER | F | 16/06/21 | 12/04/23 | 665 |

| | | | | | | |
|---|---|---|---|---|---|---|
| 81297 | Bernese Mountain Dog | CLEAR | M | 18/11/21 | 18/04/23 | 516 |
| 81334 | Rottweiler | CLEAR | F | 14/07/21 | 17/04/23 | 642 |
| 81342 | Bernese Mountain Dog | AFFECTED | F | 07/07/18 | 19/04/23 | 1747 |
| 81364 | German shepherd | CARRIER | F | 14/04/22 | 17/04/23 | 368 |
| 81426 | Australian shepherd | CARRIER | F | 20/12/19 | 20/04/23 | 1217 |
| 81446 | Miniature poodle | CLEAR | F | 24/08/21 | 22/04/23 | 606 |
| 81450 | Toy poodle | CLEAR | F | 13/09/21 | 22/04/23 | 586 |
| 81477 | Czechoslovakian wolfdog | CLEAR | F | 07/08/19 | 20/04/23 | 1352 |
| 81587 | Wire Fox terrier | AFFECTED | M | 20/03/16 | 01/05/23 | 2598 |
| 81608 | Border collie | CLEAR | F | 22/08/20 | 29/04/23 | 980 |
| 81685 | Poodle | CLEAR | F | 08/08/22 | 30/04/23 | 265 |
| 81719 | Bernese Mountain Dog | CLEAR | F | 14/05/20 | 03/05/23 | 1084 |
| 81720 | Bernese Mountain Dog | CLEAR | M | 16/07/21 | 03/05/23 | 656 |
| 81744 | Australian shepherd | CLEAR | M | 25/04/22 | 04/05/23 | 374 |
| | | | | | | |
| | | | | | | |
| | | | | | | |
| | | | | | | |
| | | | | | | |
| | | | | | | |
| | | | | | | |
| | | | | | | |
| | | | | | | |
| | | | | | | |
| | | | | | | |
| | | | | | | |
| | | | | | | |
| | | | | | | |
| | | | | | | |
| | | | | | | |
| | | | | | | |
| | | | | | | |
| | | | | | | |
| | | | | | | |
| | | | | | | |
